# Unveiling the dynamic drivers: phase separation’s pivotal role in stem cell biology and therapeutic potential

**DOI:** 10.1186/s13287-025-04403-5

**Published:** 2025-05-30

**Authors:** Pei Lin, Yunfan Lin, Ye Lu, Xu Chen, Zihao Zhou, Xinyuan Zhao, Li Cui

**Affiliations:** 1 Stomatological Hospital, School of Stomatology, Southern Medical University, Guangzhou, 510280 Guangdong China; 2 School of Dentistry, University of California, Los Angeles, Los Angeles, CA 90095 USA

**Keywords:** Stem cell biology, Phase separation, Therapeutic potential

## Abstract

Phase separation is fundamental for cellular organization and function, profoundly impacting a range of biological processes from gene expression to cellular signaling pathways, pivotal in stem cell biology. This review explores the primary types of phase separation and their mechanisms, emphasizing how phase separation is integral to maintaining cellular integrity and its significant implications for disease progression. It elaborates on current insights into how phase separation influences stem cell biology, discussing the challenges in translating these insights into practical applications. These challenges stem from the complex dynamics of phase separation, the need for advanced imaging techniques, and the necessity for real-time, in situ analysis within living systems. Addressing these challenges through innovative methodologies and gaining a deeper understanding of the molecular interactions that govern phase separation in stem cells are essential for developing precise, targeted therapies. Ultimately, advancing our understanding of phase separation could transform stem cell-based therapeutic approaches, opening up novel strategies for disease treatment and advancements in regenerative medicine.

## Introduction

Phase separation in biological systems is a pivotal process where a homogeneous cellular mixture spontaneously separates into distinct compartments or phases with different compositions and properties. This phenomenon is crucial for organizing intracellular environments without the need for membrane-bound structures, enabling the formation of membrane-less organelles like nucleoli, stress granules, and signaling complexes [1–3]. Biologically, phase separation is driven by interactions among proteins, nucleic acids, and other macromolecules, influenced by factors such as concentration, temperature, and the physicochemical properties of the cellular environment. The ability to dynamically form and dissolve these compartments allows cells to respond swiftly to environmental changes and maintain homeostasis efficiently. Importantly, phase separation provides a versatile and energy-efficient mechanism for spatial and temporal control within cells, facilitating complex biochemical processes critical for cell function and survival [4–6]. For instance, WNK1 kinases form membraneless condensates via their disordered C terminus, activating SLC12 cotransporters to restore cell volume [7]. Additionally, phase separation regulates T cell receptors (TCR) clustering and signaling by mediating CD3ε and Lck condensation into signalosomes. Lck phosphorylates CD3ε, shifting its binding to Csk, causing signalosome dissolution. This dynamic condensation-dissolution mechanism modulates T cell activation and function, highlighting phase separation’s role in receptor signaling [8].

Stem cells are distinguished by their capacity to differentiate into diverse cell types and their self-renewal properties, positioning them as a cornerstone in the treatment of a wide array of disease. This therapeutic approach leverages different types of stem cells, including embryonic stem cells (ESCs), which have pluripotent capabilities, and adult stem cells, such as mesenchymal stem cells (MSCs), which are multipotent. Increasing evidence have shown that phase separation influences key regulatory pathways and gene expression profiles essential for stem cell biology. For instance, in pluripotent stem cells, loss of LIN28 leads to nucleolar phase separation defects, causing nucleolar stress and activating a 2-cell/4-cell-like transcriptional program [9]. Therefore, elucidating the intricate relationship between phase separation and stem cell biology not only provides deeper insights into the fundamental mechanisms of cell regulation but also opens up new avenues for enhancing the efficacy and specificity of stem cell-based therapy.

In this review, several critical aspects of phase separation within cellular biology and regenerative medicine are highlighted. Initially, the types of phase separation and their formation mechanisms are outlined. Following this, the essential role of phase separation in maintaining cellular integrity and its significant implications in disease progression are analyzed. Importantly, this review is the first to critically examine and summarize the role of phase separation in regulating stem cell biology. Finally, the review addresses the current shortcomings in phase separation research related to stem cells and examines the challenges of applying phase separation in stem cell-based therapies.

## Major types of phase separation

Biological systems exhibit two primary forms of phase separation: liquid–liquid phase separation (LLPS) and liquid–solid phase separation. LLPS, driven by the minimization of free energy from favorable biomolecular interactions, results in the demixing of a homogeneous solution into two immiscible liquid phases [2, 10]. This process is essential for forming membrane-less organelles such as nucleoli, stress granules, and P-bodies [11]. The nucleolus is involved in rRNA synthesis and ribosome assembly, stress granules regulate mRNA during cellular stress, and P-bodies handle mRNA decay and storage. For example, stress granules assemble via LLPS driven by G3BP1, which acts as a molecular switch to initiate RNA-dependent LLPS [12]. Additionally, under stress, SQSTM1/p62 droplets undergo LLPS to form enlarged p62-dependent P-bodies by interacting with DDX6. These P-bodies recruit ASC, assemble the NLRP3 inflammasome, and trigger inflammation-associated cytotoxicity [13].

**Fig. 1:**
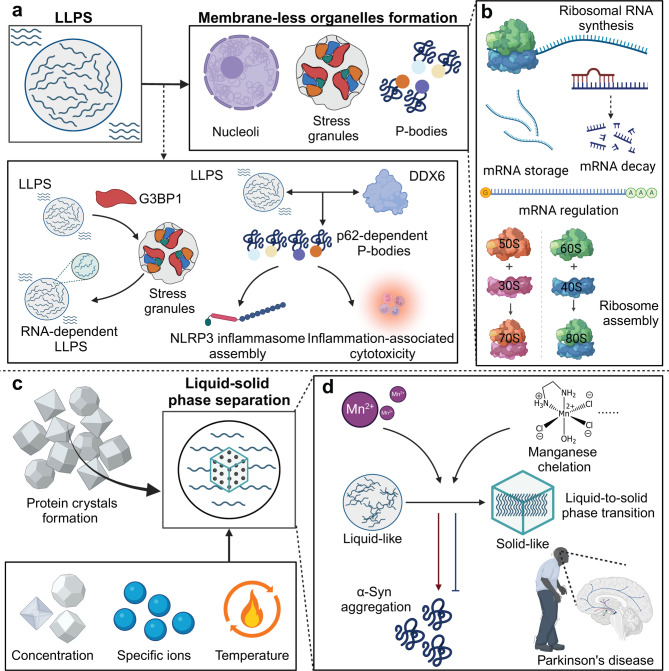
Schematic representation of major types of phase separation and their biological consequences. (**a**) LLPS and membrane-less organelle formation. LLPS enables the formation of membrane-less organelles, such as nucleoli, stress granules, and P-bodies, allowing cells to compartmentalize functions without the need for membranes. In stress granules, RNA-dependent LLPS brings together proteins like G3BP1 to promote their assembly and can also drive the formation of the NLRP3 inflammasome, potentially causing inflammation-related cell damage. Additionally, DDX6 plays a key role in forming P-bodies by interacting with p62, which helps regulate mRNA storage and degradation. (**b**) LLPS in regulating Ribosomal RNA synthesis and mRNA regulation. LLPS plays a pivotal role in ribosome biogenesis by promoting ribosomal RNA synthesis, mRNA storage, decay, and ribosome assembly, thus maintaining cellular protein production and homeostasis. (**c**) Liquid-solid phase separation and protein crystallization. Liquid-solid phase separation governs the formation of protein crystals, regulated by factors such as concentration, specific ions, and temperature. (**d**) Disease-associated liquid-to-solid phase transitions. In disease contexts, such as Parkinson’s disease, Mn²⁺ can induce a liquid-to-solid phase transition in proteins like α-synuclein, leading to pathological aggregation. Manganese chelation offers a potential strategy to prevent these transitions and mitigate disease progression

Liquid–solid phase separation involves the crystallization or precipitation of molecules from a liquid phase into a solid phase, as seen in protein crystal formation in cell vacuoles. This kind of separation is driven by changes in concentration, temperature, and the presence of specific ions or ligands. For instance, Mn²⁺ accelerates α-Syn aggregation by promoting its liquid-to-solid phase transition, directly forming solid-like condensates from soluble monomers. Notably, manganese chelation can reverse aggregation during phase transition, but not after maturation, highlighting Mn²⁺’s role in α-Syn phase separation and Parkinson’s disease pathology [14] **(**Fig. 1**)**.

## Mechanisms for forming phase separation

At the molecular level, phase separation is orchestrated by a complex interplay of interactions among biomolecules, leading to the formation of dynamic and functional compartments within cells. A central feature facilitating phase separation is the intrinsic disorder and multivalency of certain proteins and nucleic acids [15–17]. For example, BRD4 and MED1, enriched at super-enhancers, form nuclear condensates with liquid-like properties, driven by their IDRs. These condensates compartmentalize and concentrate the transcription apparatus, highlighting IDRs’ role in regulating gene expression critical for cell identity [18].

Additionally, phase separation is driven by weak, non-covalent interactions, including hydrogen bonding, electrostatic interactions, π-π stacking, and hydrophobic interactions. These weak interactions collectively contribute to the formation and stabilization of phase-separated compartments [19]. The low-complexity domain of FUS drives LLPS through multivalent interactions without traditional secondary structures, as NMR, Raman spectroscopy, and molecular simulations reveal that hydrogen bonding, π/sp2 interactions, and hydrophobic forces—particularly involving tyrosine and glutamine residues—stabilize the densely packed liquid phase [20].

Importantly, post-translational modifications (PTMs) of proteins, such as phosphorylation, methylation, and ubiquitination, can modulate phase separation by altering the interaction landscape [21, 22]. For instance, phosphorylation of Ki-67 during mitosis enhances LLPS by generating alternating charge blocks, promoting chromosome periphery formation. Conversely, phosphorylation of NPM1 reduces charge blockiness, suppressing LLPS and leading to nucleolar dissolution, demonstrating how phosphorylation modulates organelle dynamics through phase separation [23]. Interestingly, SUMOylation of RNF168 induces LLPS, limiting its recruitment to DNA damage sites and impairing repair, while SENP1-mediated deSUMOylation prevents LLPS, enhancing DNA repair efficiency [24].

Moreover, RNA molecules act as critical scaffolds in phase separation, providing a structural framework that facilitates the assembly of protein-RNA complexes [25]. The secondary structures of RNA, such as stem-loops and G-quadruplexes, create binding sites for multiple RNA-binding proteins, promoting multivalent interactions. In the nucleolus, rRNA serves as a scaffold for the assembly of ribosomal proteins and other nucleolar components, driving the formation of distinct nucleolar subdomains through LLPS [26]. For instance, in the DNA damage response, TopBP1 interacts with pre-rRNA at DNA damage sites to promote LLPS for efficient repair [27].In addition, SNHG9, a lipid-associated lncRNA, promotes LLPS of LATS1, inhibiting its activity in the Hippo pathway and enhancing YAP signaling, which correlates with breast cancer progression [28].

**Fig. 2:**
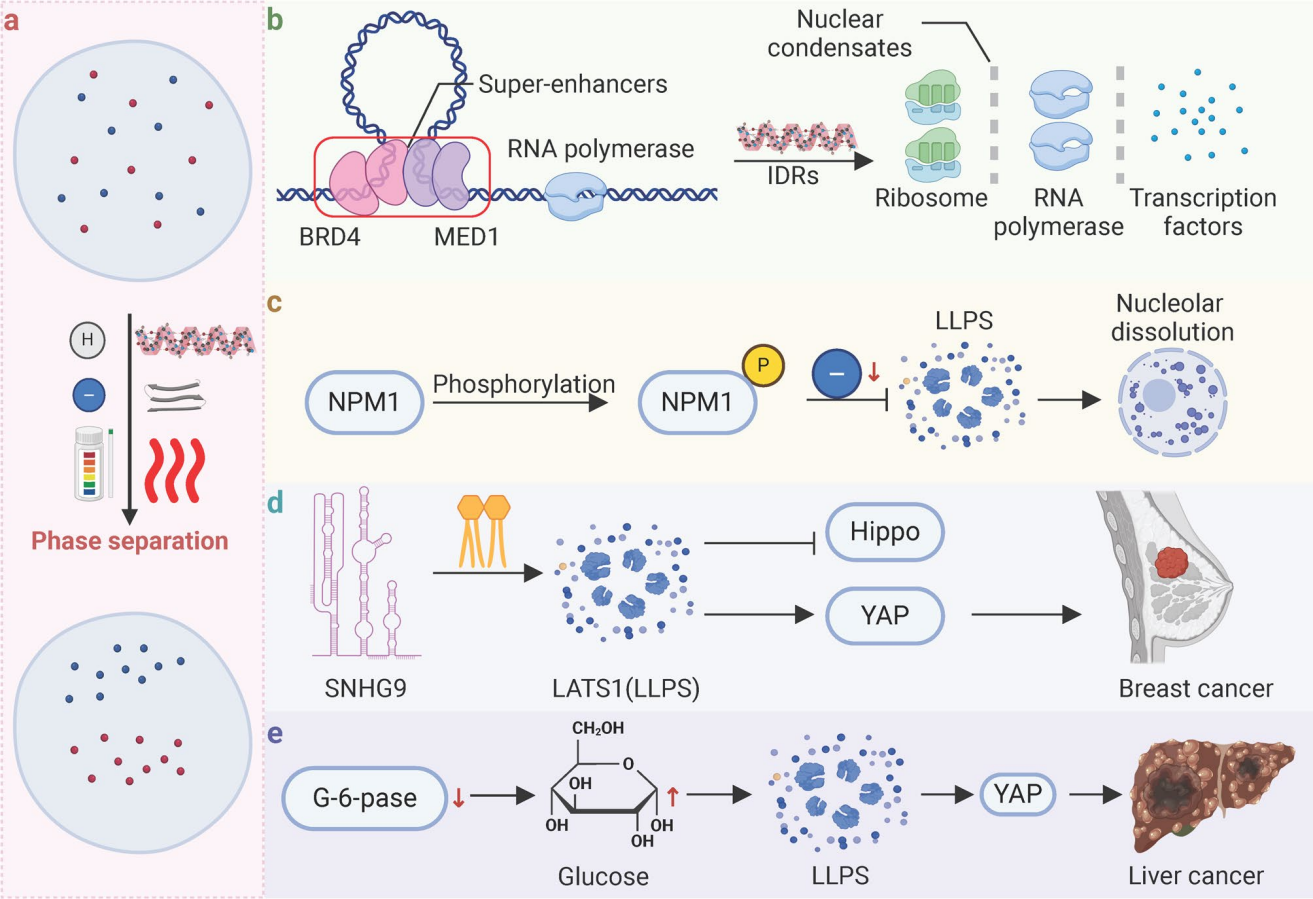
The mechanisms for phase separation formation. (**a**) Triggers of phase separation. Phase separation can be triggered by changes in factors such as pH and temperature, leading to the formation of distinct liquid compartments. (**b**) Example of IDRs driven LLPS. Transcriptional co-activators BRD4 and MED1, enriched at SEs, can undergo phase separation via their IDRs, forming droplets that compartmentalize and concentrate the transcription apparatus from nuclear extracts, including ribosomes, RNA polymerase, and transcription factors, to regulate key cellular gene expression. (**c**) Example of how modulate phase separation. Phosphorylation of NPM1 regulates its phase separation behavior, leading to nucleolar dissolution upon nucleophosmin’s dissociation from nuclear condensates. (**d**) Example of RNA molecules driven LLPS. In cancer, SNHG9 and LATS1 undergo LLPS, influencing the Hippo pathway and YAP activation, which promotes breast cancer progression. (**e**) Example of biomolecules concentration driven LLPS. The downregulation of G6PC in the liver leads to glucose accumulation within cells, which undergoes LLPS, activating YAP and promoting malignant transformation of liver cells

Notably, phase-separated compartments typically form when the concentration of involved biomolecules exceeds specific thresholds, triggering droplet nucleation and growth [29]. In liver cells, glycogen accumulation due to reduced glucose-6-phosphatase expression induces LLPS, sequestering Hippo kinases Mst1/2 and activating Yap to promote tumorigenesis [30]. Environmental factors such as pH, ionic strength, and temperature profoundly influence phase separation by altering electrostatic interactions and droplet stability [1]. For instance, elevated salt and temperature induce RALF-pectin phase separation, forming condensates that promote receptor clustering, endocytosis, and plant stress recovery [31] **(**Fig. 2**)**.

## Phase separation is crucial for cell function and disease development

By facilitating the segregation and concentration of specific proteins, RNA, and other biomolecules, phase separation ensures the precise control of signaling pathways, gene expression, and metabolic processes that are essential for regulating stem cell biology. For instance, the mechanism by which promyelocytic leukemia protein (PML) manages oxidative stress responses in ESCs through SUMO2/3 conjugation is pivotal for its function within nuclear bodies, influencing KAP1 SUMOylation to modulate the epigenetic repression of retro-elements. Loss of PML leads to the re-expression of transposable elements and the acquisition of 2-cell-like features via SUMO2 modification of DPPA2, illustrating PML’s critical role in coordinating stem cell states and its potential implications in cancer biology [32]. Additionally, during zebrafish embryogenesis, Ddx3xb undergoes LLPS via its N-terminal IDR, enhancing maternal mRNA translation. Mutations impairing phase separation hinder embryo development, demonstrating LLPS’s role in gene activation [33].

**Fig. 3:**
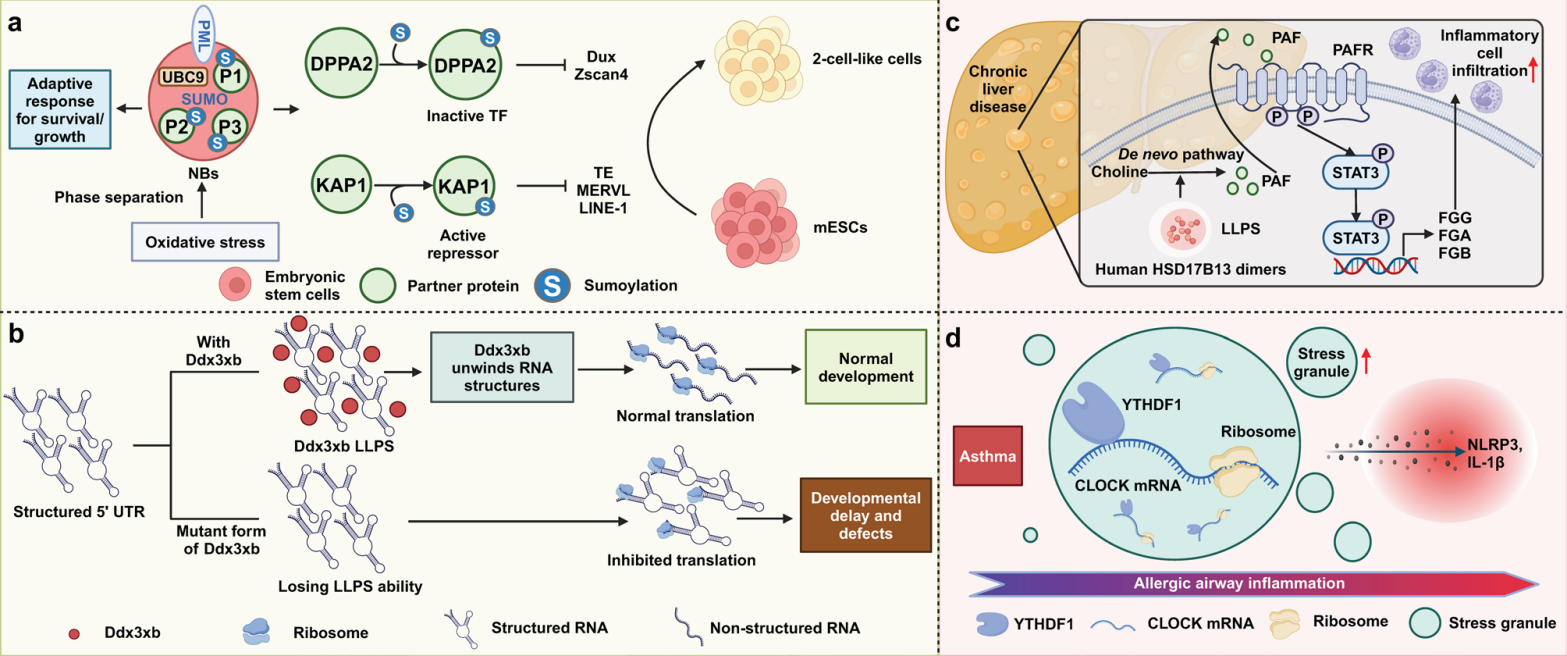
Phase separation in regulating cellular function and disease progression. (**a**) SUMOylation modulates transcription factor activity in response to oxidative stress. UBC9-mediated SUMOylation of DPPA2 and KAP1 regulates the activation of Dux and Zscan4, promoting the transition of embryonic stem cells into 2-cell-like cells. Meanwhile, SUMOylation of KAP1 preserves its role as an active repressor in mESCs. This post-translational modification plays a critical role in maintaining the balance of cell fate decisions under oxidative stress. (**b**) Ddx3xb facilitates normal translation by unwinding structured 5′ UTRs, allowing ribosomes to access mRNAs, which supports normal development. In the absence of Ddx3xb, structured RNAs block ribosome binding, leading to inhibited translation and resulting in developmental delays and defects. (**c**) In chronic liver disease, PAFR activation via the de novo choline pathway leads to inflammatory cell infiltration. LLPS drives the assembly of PAF, which, together with activated STAT3, upregulates the expression of fibrinogen components (FGG, FGA, FGB), contributing to disease progression. (**d**) In asthma, stress granule formation is elevated, enhancing NLRP3 inflammasome activity and increasing pro-inflammatory cytokines IL-1β. The phase separation of YTHDF1 with *CLOCK* mRNA within stress granules contributes to allergic airway inflammation, linking RNA metabolism to immune responses

Abnormal phase separation is increasingly recognized as a critical factor in the development and progression of various diseases [34–37]. Notably, HSD17B13 forms LLPS around lipid droplets, enhancing its enzymatic function and increasing platelet activating factor biosynthesis. This promotes fibrinogen synthesis and leukocyte adhesion, exacerbating liver inflammation [38]. Likewise, YTHDF1, an m6A reader highly expressed in asthmatic patients, undergoes allergen-enhanced LLPS to form complexes with *CLOCK* mRNA. This boosts CLOCK translation, activates NLRP3 inflammasome, and increases IL-1β secretion, driving airway inflammation. Deleting CLOCK abolishes these effects, highlighting YTHDF1 as a therapeutic target for allergic airway inflammation [39]. Moreover, thousands of disease-linked genetic variants in intrinsically disordered protein regions impact phase separation, altering biomolecular condensates like the nucleolus. Variants in these regions can disrupt nucleolar function. Frameshift mutations in HMGB1, for instance, lead to brachyphalangy, polydactyly, and tibial aplasia syndrome by changing HMGB1 phase separation and nucleolar partitioning [34] **(**Fig. 3**)**.

## The effect of phase separation on stem cell biology

### The effect of phase separation on stem cell fate determination

Recent advances reveal phase separation plays a significant role in controlling stem cell fate, where it orchestrates the formation of compartmentalized environments crucial for modulating signaling pathways that dictate stem cell identity and lineage commitment. Insights into this mechanism not only deepen our understanding of stem cell biology but also provide a foundation for novel therapeutic approaches. For instance, CINAP acts as a negative regulator of YAP1 during ESC differentiation by interacting with NEDD4 to prevent its phase separation and cytoplasmic condensation. This interaction halts YAP1 compartmentalization and facilitates its activation, crucial for directing cell fate decisions. Depletion of CINAP promotes NEDD4 condensates, leading to unintended YAP1 activation and disrupting endoderm differentiation, highlighting the critical role of phase separation in regulating stem cell fate and early embryonic development [40]. Similarly, the destruction complex for Wnt signal transduction undergoes phase separation, nucleated by the centrosome, which enhances β-catenin processing. This localization consolidates destruction complex components into a single reaction site, effectively controlling β-catenin stability and preventing Wnt-induced differentiation of embryonic stem cells into mesoderm. This mechanism underscores the integration of Wnt signaling with cell cycle regulation and highlights the importance of nucleators in organizing biomolecular condensates to modulate stem cell fate decisions [41]. Notably, LIN28A, localized in the nucleolus, facilitates liquid-liquid phase separation in ESCs. Its RNA binding domains and intrinsically disordered regions drive condensate formation with nucleolar proteins. Mutations in these regions disrupt LIN28A’s subcellular localization and its phase separation, impairing nucleolar function and cell fate decisions. This highlights LIN28A’s pivotal, non-canonical role in nucleolar dynamics and stem cell state transitions through phase separation mechanisms [42]. Additionally, Cpeb1b-mediated cytoplasmic polyadenylation in zebrafish, facilitated by phase separation in liquid-like condensates, specifically enhances Hedgehog signaling to drive hematopoietic stem and progenitor cell specification. This effect is achieved through selective interactions with shha mRNA within the condensates, emphasizing the role of phase separation in regulating translation and subsequent cell fate determination during early development [43]. Moreover, liquid-liquid phase separation of the m^6^A “reader” protein YTHDF1 regulates spermatogonial stem cell transdifferentiation into neural-like cells by modulating the IκB-NF-κB-CCND1 axis. Disruption of YTHDF1 LLPS or NF-κB pathway inhibits this process, while overexpression of tau-YTH fusion protein, enhancing tau LLPS, restores it by reactivating the axis. These findings highlight LLPS’s critical role in controlling cell fate and underscore its potential therapeutic relevance in neurological disease treatment [44]. Interestingly, activating SHP2E76K mutation in MSCs triggers malignant transformation by enhancing mitochondrial metabolism via complexes I and III. Liquid-liquid phase separation of SHP2, promoting its dissociation from these complexes, drives their hyperactivation. Inhibiting SHP2 LLPS curbs this hyperactivation, offering a therapeutic angle for targeting SHP2-associated malignancies. This delineates a role for phase separation in the pathological alteration of MSC fate [45].

**Fig. 4:**
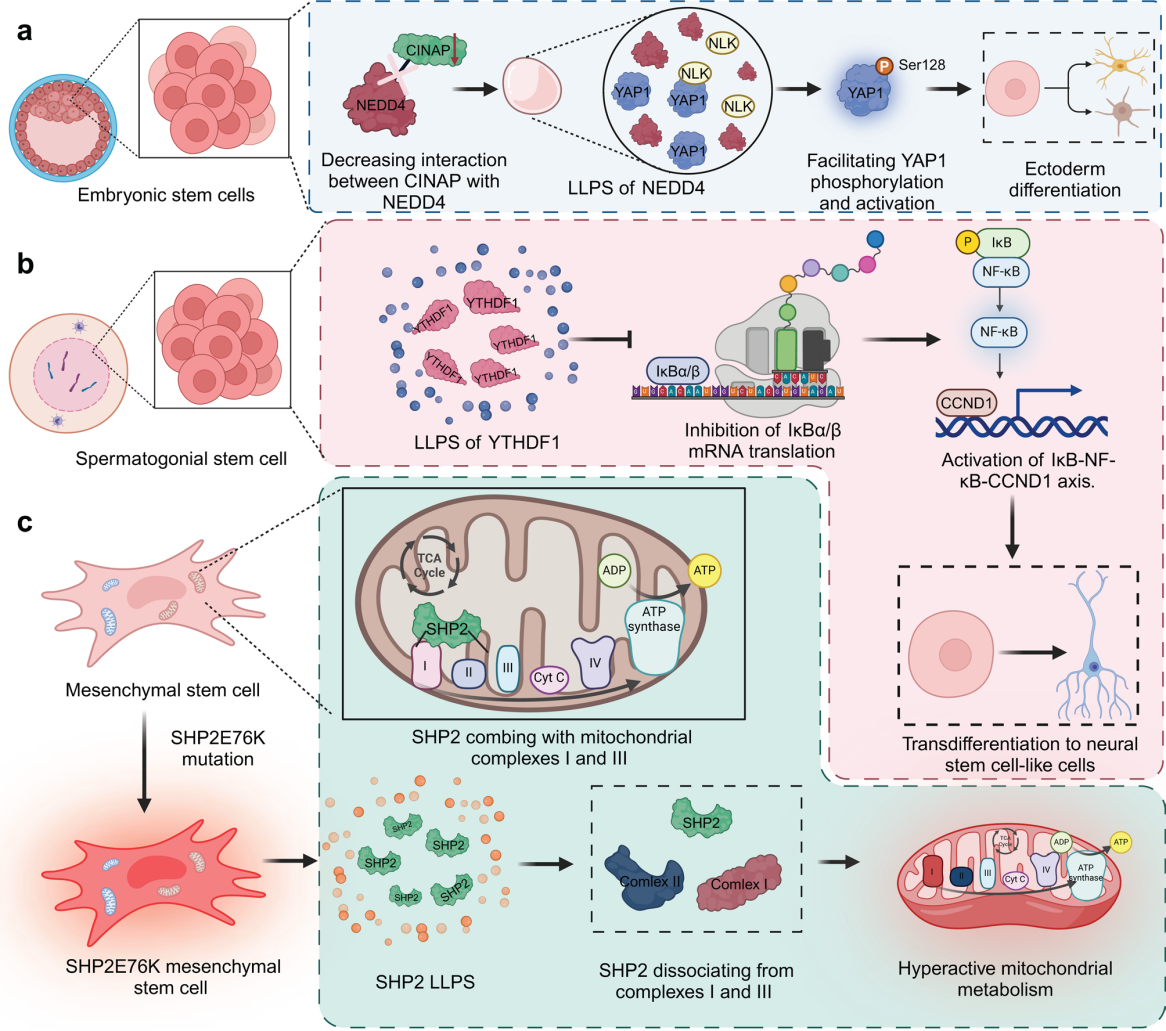
The effect of phase separation on stem cell fate determination. (**a**) In ESCs, LLPS of NEDD4 is regulated by a reduction in the interaction between CINAP and NEDD4. This phase separation event enhances YAP1 phosphorylation at Ser128 by NLK, activating YAP1 and driving ectoderm differentiation. (**b**) In spermatogonial stem cells, LLPS of YTHDF1 plays a key role in inhibiting the translation of IκBα/β mRNA, which results in the activation of the IκB-NF-κB-CCND1 axis. This activation contributes to the transdifferentiation of spermatogonial stem cells into neural stem-like cells. (**c**) In MSCs, the SHP2E76K mutation induces LLPS of SHP2. This mutation alters SHP2’s interaction with mitochondrial complexes I and III, leading to dissociation from these complexes and promoting hyperactive mitochondrial metabolism which is closely linked to the transdifferentiation process of MSCs

In addition to animal stem cells, phase separation has also been demonstrated to play a critical role in mediating plant stem cell fate determination. In the Arabidopsis root stem cell niche, transcription factors WOX5 and PLTs, particularly PLT3 with its prion-like domains, regulate quiescence and fate of columella stem cells through complex formation and recruitment to subnuclear microdomains, likely via phase separation. This spatial organization within nuclear bodies underscores a critical mechanism where mutual regulation of WOX5 and PLTs at transcriptional and protein interaction levels determines stem cell behavior and root development [46] **(**Fig. 4**)**.

### The effect of phase separation on stem cell differentiation

Stem cell differentiation is the process by which pluripotent or multipotent cells, inherently capable of self-renewal, commit to specific lineages and develop distinct morphological and functional characteristics typical of mature cell types. This transition is orchestrated by a complex interplay of genetic and epigenetic factors, coupled with cues from the cellular microenvironment. As stem cells differentiate, they undergo profound alterations in gene expression, protein synthesis, and cellular architecture, enabling them to perform specialized functions within an organism [47]. The hubs formed by phase separation can enhance or inhibit specific signaling pathways [48–50], thereby directing the developmental trajectory of stem cells. Understanding phase separation thus provides valuable insights into the regulatory mechanisms of stem cell fate and opens potential avenues for advancing regenerative medicine strategies.

Chromobox (CBX) proteins function within the Polycomb repressive complex 1 (PRC1) to modulate chromatin architecture through phase-separated condensates. This process is critical for repressing stem cell-active genes and guiding the differentiation of stem cells by affecting chromatin compaction and gene accessibility. In vivo studies reveal that CBX2, through its nonenzymatic actions, regulates spermatogonial stem cell differentiation in male germline by forming phase-separated condensates that compact chromatin and repress stem cell-active genes. Specifically required for the differentiation into A1 spermatogonia, CBX2’s ability to modulate chromatin architecture via phase separation is essential for the long-term maintenance of male germ cells, highlighting a pivotal role beyond histone modification [51]. Similarly, introducing a chromatin compaction and phase separation domain into CBX7 in ESCs disrupts their differentiation, impairing embryoid body and neural progenitor formation while maintaining inappropriate Polycomb binding at neural-specific loci. This suggests that the ability to compact chromatin and undergo phase separation is crucial for the Polycomb group’s role in transitioning from pluripotency to differentiated states, highlighting phase separation as a key epigenetic mechanism in stem cell lineage specification [52]. In addition, CBX7C, a splicing isoform of CBX7, acts as an epigenetic repressor in ESCs, coordinating with PHC2 to guide PRC1 complex assembly at canonical targets. This interaction drives the formation of Polycomb bodies, with phase separation dynamics influenced by protein concentration: low levels promote the creation of highly mobile functional aggregates, while high concentrations lead to larger, less mobile structures. Altering CBX7C levels affects stem cell differentiation, highlighting phase separation’s role in stem cell regulation and PRC1 activity modulation [53].

**Fig. 5:**
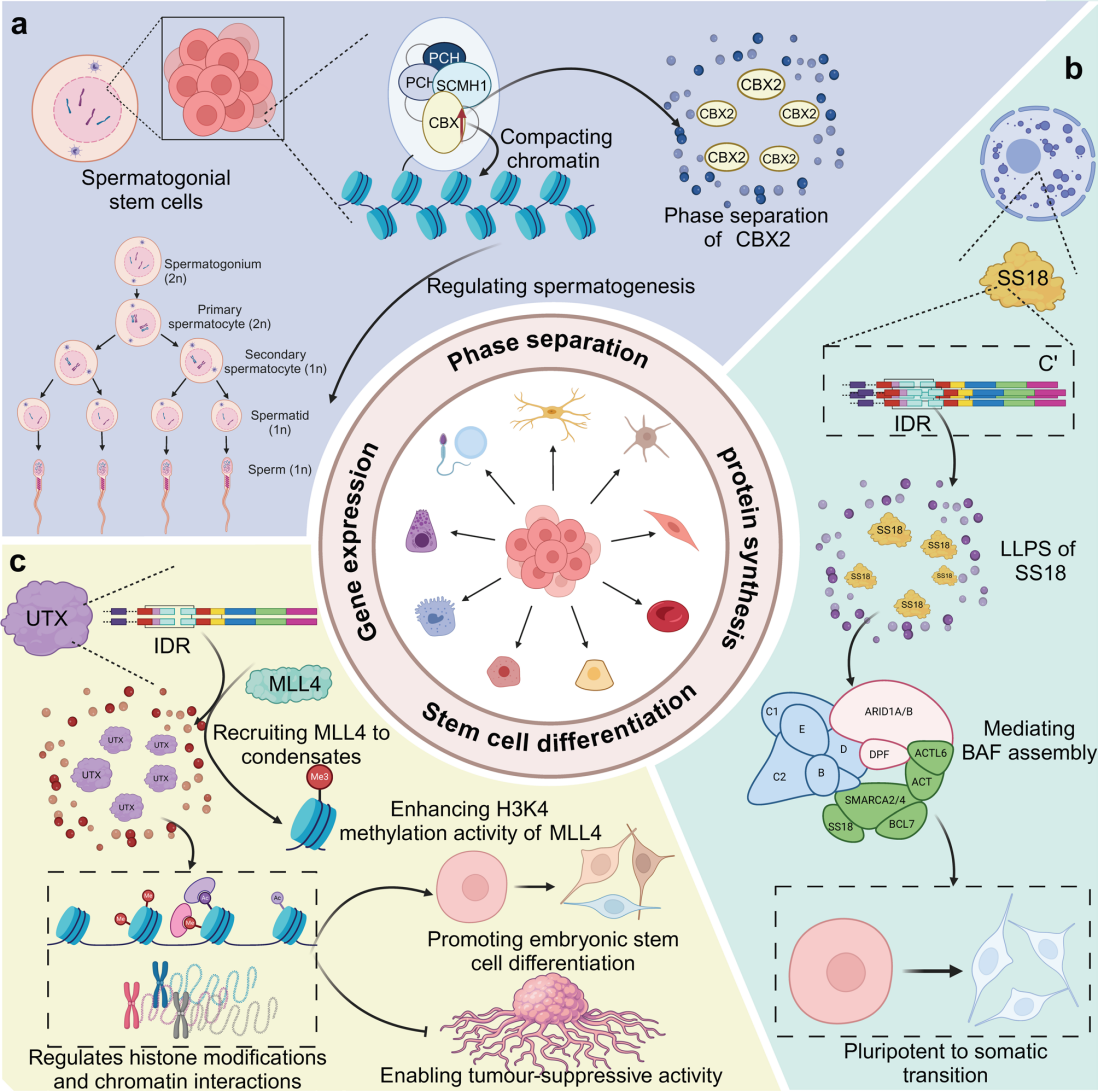
The effect of phase separation on stem cell differentiation. (**a**) Phase separation of CBX2 in spermatogonial stem cells drives chromatin compaction, which is essential for regulating spermatogenesis. CBX2 forms a phase-separated condensate by interacting with PCH, PC, and SCMH1, facilitating chromatin condensation and gene regulation during the transition from spermatogonia to mature sperm cells. (**b**) SS18 undergoes LLPS through its IDR, mediating the assembly of the BAF complex, which plays a critical role in the pluripotent to somatic cell transition. (**c**) The IDR of UTX recruits MLL4 to form phase-separated condensates, enhancing MLL4’s H3K4 methylation activity. This process regulates histone modifications and chromatin interactions, promoting embryonic stem cell differentiation and enabling tumor-suppressive activity. At the core, phase separation influences essential biological processes such as gene expression, protein synthesis, thereby playing a crucial role in stem cell differentiation

SS18 regulates the pluripotent to somatic transition (PST) in mammalian development by forming nuclear condensates via a C-terminal intrinsically disordered region (IDR) rich in tyrosine. The IDR is necessary but not sufficient for PST, requiring an N-terminal 70aa segment to interact with the BAF complex. SS18-mediated BAF assembly through phase separation is essential for PST, highlighting a unique tyrosine-based mechanism in regulating stem cell differentiation [54]. In addition, in zebrafish models, Tet2/3 and Sall4 regulate pharyngeal cartilage development via a TET-BMP-Sall4 axis, critical for craniofacial microsomia pathogenesis. Loss of Tet2/3 disrupts chondrocyte differentiation by impairing BMP signaling, while Sall4 activates bmp4 expression through co-phase separation with Tet2/3, enhancing 5mC oxidation at the bmp4 promoter. This molecular mechanism underscores phase separation’s role in controlling gene expression and developmental pathways in craniofacial disorders [55]. Moreover, TAZ interacts with Smad7 and β-catenin to suppress muscle-specific gene expression, including the creatine kinase muscle gene and myogenin. Ectopic TAZ expression inhibits β-catenin activity and myogenic differentiation, while its depletion enhances promoter activation. TAZ localization shifts from nuclear speckles to the cytoplasm upon differentiation, facilitated by Ser89 phosphorylation. Moreover, TAZ demonstrates liquid-liquid phase separation properties, suggesting a regulatory mechanism in stem cell differentiation through spatial compartmentalization [56]. Likewise, Dact1 is upregulated during myogenesis, facilitating terminal differentiation, cell cycle withdrawal, and cell fusion. In human muscle pathologies, Dact1 expression is altered. Bioinformatic analysis reveals Dact1’s long intrinsically disordered regions, enabling liquid-liquid phase separation and nuclear aggregate formation [57]. Furthermore, the homeodomain transcription factor Prospero (Pros)/Prox1 drives neuronal differentiation by inducing heterochromatin condensation via liquid-liquid phase separation. In Drosophila neural precursors, Pros retains at H3K9me3^+^ pericentromeric regions during mitosis, recruiting heterochromatin protein 1 (HP1) into phase-separated condensates to compact heterochromatin. This process ensures cell-cycle exit and terminal differentiation. Mammalian Prox1 similarly employs this “mitotic-implantation-ensured heterochromatin condensation” strategy, highlighting a conserved mechanism where LLPS-mediated chromatin remodeling secures neuronal differentiation [58]. Interestingly, investigations into GelMA scaffolds reveal that increases in elastic modulus enhance osteogenic differentiation in hBMSCs, associated with altered YAP, TAZ, and TEAD expression and their assembly into phase-separated condensates sensitive to 1’6-hexanediol. In vivo, higher modulus GelMA better supports new bone formation, highlighting substrate stiffness and liquid-liquid phase separation as critical factors in regulating stem cell function and bone regeneration [59]. UTX, a histone H3K27 demethylase, exerts its biological function through phase separation via its core IDR. This region forms liquid condensates critical for tumor suppression and ESC differentiation by recruiting MLL4 and enhancing H3K4 methylation [60]. Importantly, MAGE-B2 enhances cellular stress tolerance by inhibiting SG formation through translational repression of G3BP. By reducing G3BP levels below the threshold for phase separation, MAGE-B2 prevents SG initiation. Knockout of the MAGE-B2 ortholog or G3BP1 overexpression increases male germline sensitivity to heat stress, highlighting MAGE-B2’s role in protecting spermatogenesis [61] **(**Fig. 5**)**.

### The effect of phase separation on stem cell reprogramming

Stem cell reprogramming represents a transformative advance in regenerative medicine, enabling the conversion of differentiated cells back to a pluripotent state. This process effectively resets the cellular clock, endowing adult cells with the ability to differentiate into any cell type, akin to embryonic stem cells. The significance of this technique lies in its potential to provide patient-specific therapies, eliminating the ethical and immunological complications associated with embryonic stem cells. Remarkably, during somatic cell reprogramming, extensive TAD reorganization correlates with gene transcription and cell identity changes. OCT4 phase-separated condensates drive this TAD reorganization through concentrated chromatin loops. Disruption of OCT4 phase separation impairs TAD reorganization and reprogramming, but can be rescued by fusing an intrinsically disordered region (IDR) to OCT4. Using TAD reorganization-based multiomics analysis, key reprogramming regulators were identified, highlighting the crucial role of OCT4-mediated phase separation in cellular reprogramming [62].

### The effect of phase separation on stem cell self-renewal and pluripotency

The attributes of self-renewal and pluripotency in stem cells are critical to their function and potential applications in developmental biology, regenerative medicine, and disease modeling. Self-renewal refers to the ability of stem cells to undergo numerous cycles of cell division while maintaining an undifferentiated state [63, 64]. This property ensures a stable population of stem cells that can support tissue homeostasis and repair throughout the lifespan of an organism. Pluripotency, the capacity of stem cells, particularly embryonic stem cells, to differentiate into nearly any cell type within an organism, is essential for generating the diverse cellular constituents necessary during embryonic development and offers significant therapeutic potential. Recent studies have highlighted the role of phase separation in regulating self-renewal and pluripotency in stem cells. For instance, RYBP-mediated phase separation of CTCF organizes long-range chromatin interactions between A compartments, diverging from traditional loop extrusion models. An engineered system inducing CTCF phase separation in ESCs enhanced these interactions, supporting ESC self-renewal and inhibiting differentiation towards neural progenitor cells. This demonstrates a non-canonical role for CTCF in chromatin architecture, revealing phase separation as a key regulator of stem cell self-renewal through spatial chromatin organization [65]. Additionally, ASXL1 promotes paraspeckle formation and hematopoiesis through its IDR, facilitating NONO-NEAT1 interactions and NEAT1 expression. A pathogenic ASXL1 mutant lacking the IDR disrupts these processes, leading to abnormal NONO localization in hematopoietic stem and progenitor cells (HSPCs). Both NONO depletion and cytoplasmic mislocalization impair HSPC repopulating potential, highlighting ASXL1’s role in maintaining hematopoiesis via phase separation-driven paraspeckle assembly [66]. Moreover, MIP-1 and MIP-2 are novel *C. elegans* germ granule components that interact with MEG-3 to promote P granule condensation and balance their growth and localization. Containing LOTUS domains and intrinsically disordered regions, they bind and anchor GLH-1 within P granules, facilitating the coalescence of MEG-3, GLH-1, and PGL proteins. Loss of MIP-1 and MIP-2 leads to temperature-sensitive embryonic lethality, sterility, and germline defects, affecting stem cell self-renewal and gamete differentiation, underscoring their role in organizing ribonucleoprotein networks for germline development [67].

**Fig. 6:**
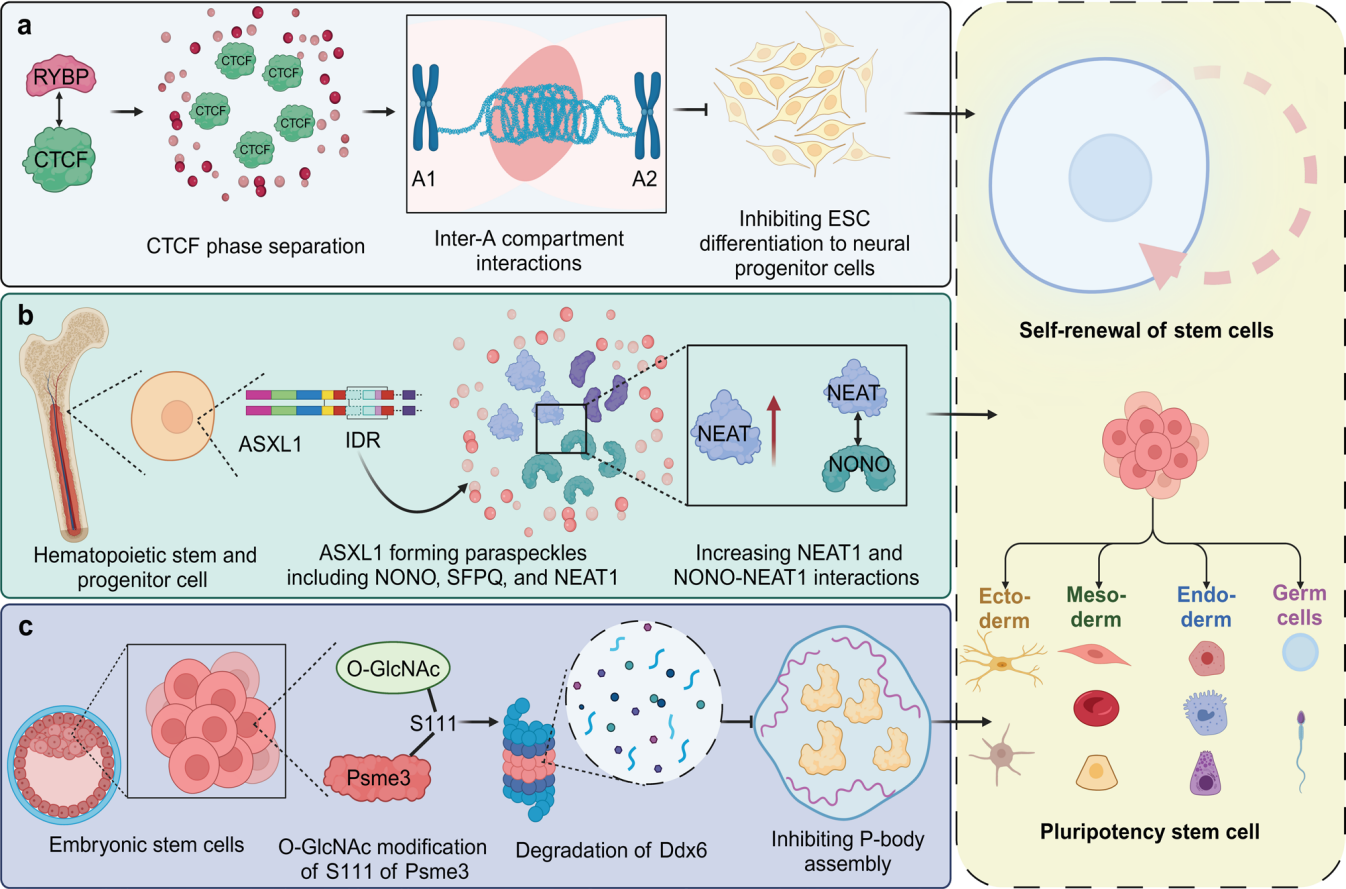
The effect of phase separation on stem cell self-renewal and pluripotency. (**a**) CTCF forms phase-separated condensates in association with RYBP, mediating interactions between A1 and A2 compartments to regulate chromatin organization. This phase separation process inhibits the differentiation of ESCs into neural progenitor cells, thereby maintaining stem cell self-renewal. (**b**) ASXL1, through its IDR, forms paraspeckles with proteins such as NONO, SFPQ, and NEAT1 in hematopoietic stem and progenitor cells. This interaction increases the formation of NONO-NEAT1 complexes, promoting paraspeckle assembly and enhancing stem cell maintenance. (**c**) O-GlcNAc modification at S111 of Psme3 in embryonic stem cells leads to the degradation of Ddx6 and inhibits P-body assembly, further supporting the self-renewal and pluripotency of stem cells by modulating RNA metabolism. Collectively, these mechanisms contribute to the balance between self-renewal and differentiation across various stem cell types

In addition to self-renewal, phase separation has been shown to critically regulate stem cell pluripotency. ABCF1 facilitates phase separation and pluripotency gene activation via multivalent interactions with SOX2 and coactivators XPC and DKC1. ABCF1’s LCD-mediated interactions are disrupted by DNA damage, highlighting its dual role in maintaining stem cell pluripotency and responding to genomic integrity threats [68]. Importantly, single-molecule microscopy reveals that genome remodeling during the transition from naïve embryonic to epiblast stem cells involves chromatin decompaction and reduced OCT4 mobility. This correlates with decreased H3K9ac marks and lowered Oct4 RNA expression. Spatial reconfiguration brings Oct4 closer to nuclear speckles and Nodal alleles, influencing pluripotency gene expression during epiblast specification, illustrating the critical interplay of genome organization and gene regulation in stem cell pluripotency [69]. Additionally, O-GlcNAcylation of Psme3 at serine 111 is crucial for maintaining ESC pluripotency by regulating phase separation through P-body homeostasis. This modification promotes Ddx6 degradation, reducing P-body assembly and sustaining the pluripotent state. Conversely, loss of Psme3 O-GlcNAcylation stabilizes Ddx6, increasing P-body levels and triggering ESC differentiation [70] **(**Fig. 6**)**.

### The effect of phase separation on stem cell senescence

Stem cell senescence is a pivotal phenomenon in the biology of aging, profoundly influencing tissue degeneration, disease progression, and the decline in regenerative capacity [71]. As stem cells age, they lose their abilities for self-renewal and pluripotency, leading to a diminished capacity to maintain tissue integrity and function. Accumulated DNA damage, oxidative stress, and metabolic shifts, often exacerbated by aging, can drive stem cells into a senescent state. This not only impacts individual health and longevity but also serves as a potential driver for various age-related diseases [72–74]. Understanding the role of phase separation in stem cell senescence offers novel insights into the mechanisms of aging and highlights potential therapeutic targets for ameliorating age-related cellular decline.

**Fig. 7:**
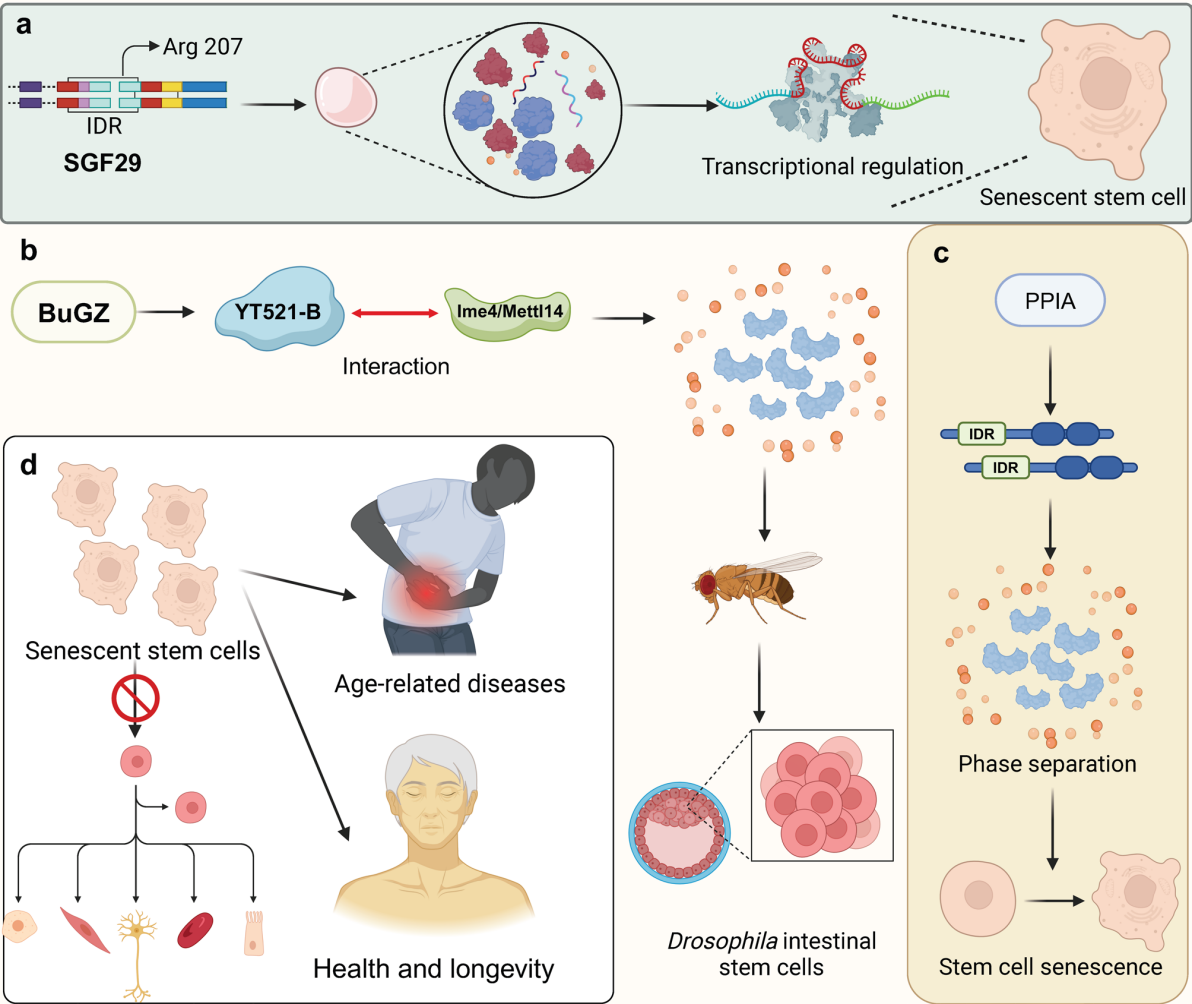
The effect of phase separation on stem cell senescence. (**a**) SGF29, through its IDR and Arg 207, undergoes phase separation to regulate transcriptional activity, regulating transcriptional activity and contributing to stem cell senescence. (**b**) The interaction between BuGZ, YT521-B, and the m^6^A methyltransferase complex components lme4/Mettl14 mediates phase separation in Drosophila intestinal stem cells, influencing stem cell dynamics. This phase separation impacts the maintenance of stem cell populations and links senescent stem cells to age-related diseases, ultimately affecting health and longevity. (**c**) PPIA, through its IDRs, also undergoes phase separation, promoting stem cell senescence. (**d**) Collectively, these mechanisms illustrate the role of phase separation in stem cell regulation, aging, and the development of age-related pathologies

Peptidyl-prolyl isomerase A (PPIA) emerges as a crucial chaperone in haematopoietic stem and progenitor cells, with its depletion linked to accelerated ageing. PPIA predominantly targets proteins with IDRs, promoting their involvement in phase separation and the formation of supramolecular, membrane-less organelles. This interaction boosts cellular stress resistance but diminishes with age due to a decline in PPIA expression, highlighting a mechanism where impaired phase separation contributes to stem cell ageing [75]. Additionally, SGF29, a SAGA complex component, forms liquid-like nuclear condensates influencing transcriptional regulation during cellular senescence in mesenchymal progenitor cells. Key to condensate formation is Arg 207 in SGF29’s disordered region, crucial for chromatin targeting and gene activation linked to senescence, such as CDKN1A. While essential for precise chromatin engagement and co-activator recruitment, SGF29 condensates alone do not suffice for H3K4me3 binding or full transactivation, underscoring phase separation’s role in modulating transcriptional landscapes during aging [76]. Moreover, BuGZ, a coacervating mitotic effector, demonstrates age- and injury-associated nuclear condensation in *Drosophila* intestinal stem cells (ISCs), enhancing ISC proliferation and impacting gut repair and longevity. The m^6^A reader YT521-B, functionally downstream of BuGZ, influences its coacervation via interactions with the m^6^A writer Ime4/Mettl14. This suggests a role for phase separation and m^6^A regulation in modulating ISC-dependent regeneration and aging, highlighting a novel avenue for potential therapeutic interventions [77] **(**Fig. 7**)**.

### The effect of phase separation on stem cell transcriptional activity

**Fig. 8:**
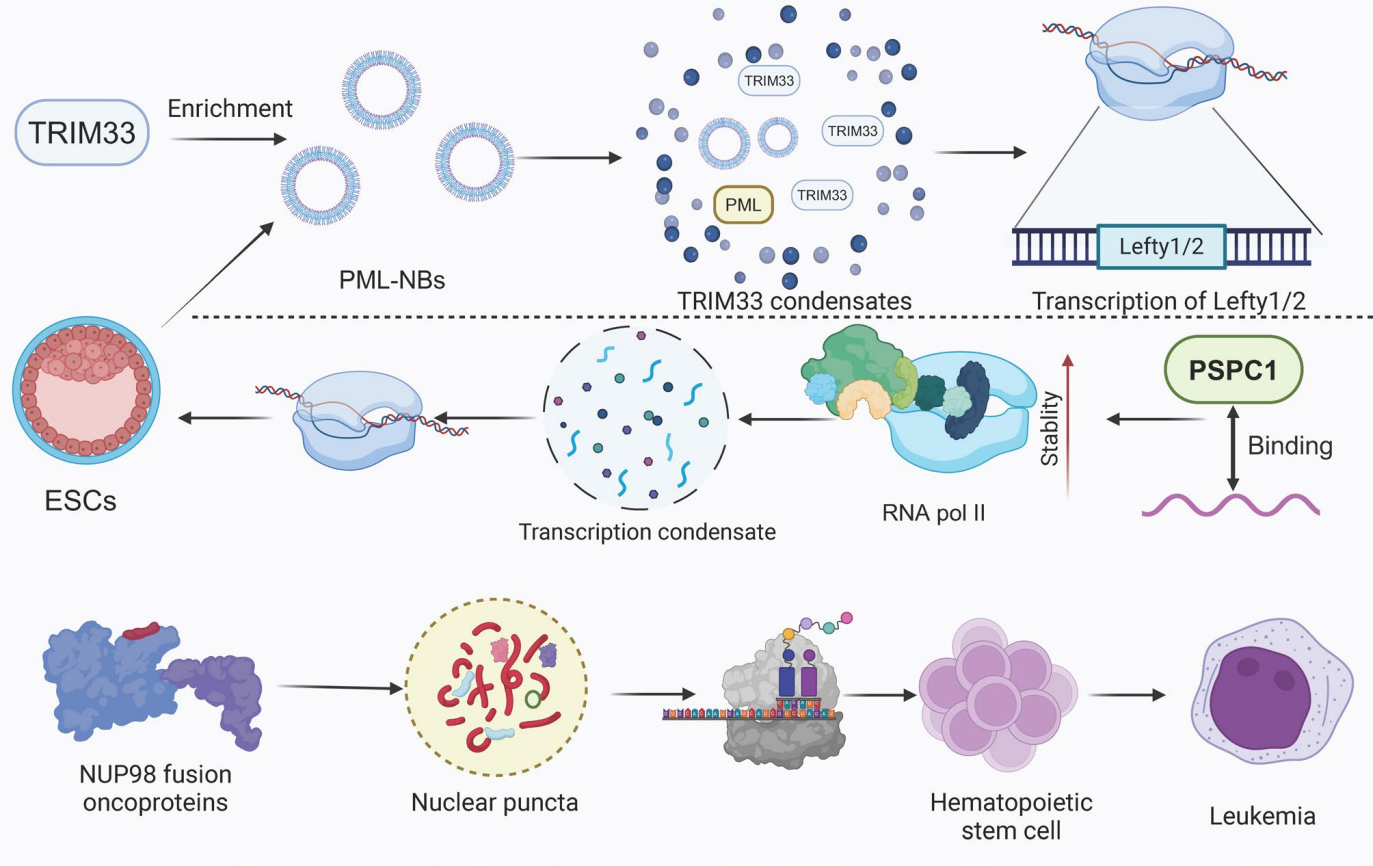
The effect of phase separation on stem cell transcriptional activity. TRIM33 forms phase-separated condensates within PML-NBs in ESCs, facilitating the transcription of *Lefty1/2*. These condensates stabilize RNA polymerase II (RNA Pol II) within transcriptional complexes, enhancing mRNA stability. Additionally, PSPC1 binds to transcriptional condensates, further supporting mRNA transcript stabilization. In hematopoietic stem cells, NUP98 fusion oncoproteins form nuclear puncta, disrupting normal transcriptional regulation and contributing to leukemia progression

Transcriptional activity within stem cells is a cornerstone of their unique identity and capabilities, underpinning the intricate balance between self-renewal and differentiation. The precise regulation of gene expression is fundamental to the ability of stem cells to respond to developmental cues and environmental stimuli, thereby dictating their fate decisions and functional diversity. TRIM33 localizes with promyelocytic leukemia nuclear bodies (PML-NBs) in ESCs to regulate Nodal signaling-mediated transcription of Lefty1/2. This interaction requires PML-NB-specific assembly, with both PML protein and PML-NB formation necessary for TRIM33’s recruitment to these gene loci. Proximity labeling confirms TRIM33’s enrichment in ESC-specific PML-NBs, highlighting how phase separation influences stem cell transcriptional regulation in a context-dependent manner [78]. In addition, RNA-binding proteins, notably PSPC1, are pivotal in connecting RNA to transcription machinery in ESCs, facilitating phase separation at transcription sites. PSPC1 harnesses RNA to stabilize RNA polymerase II (Pol II), enhancing transcription condensate formation and polymerase activity. Acute PSPC1 depletion disrupts Pol II binding and transcription, underscoring the role of RNA-mediated phase separation in promoting active transcription and maintaining stem cell transcriptional integrity [79]. Aberrations in transcriptional processes can lead to dysregulated stem cell activity, which is implicated in a variety of disorders, including cancer. For instance, NUP98 fusion oncoproteins, including NUP98-HOXA9, drive leukemia by forming nuclear puncta through liquid-liquid phase separation, involving both homotypic and heterotypic interactions. These condensates modulate transcriptional activity and transform hematopoietic stem and progenitor cells, a mechanism extendable to other leukemia-associated NUP98 fusions [80] **(**Fig. 8**)**.

### The effect of phase separation on stem cell chromatin organization and stability

Chromatin organization and stability are critical for the regulation of gene expression and the maintenance of genomic integrity in stem cells. Proper chromatin structuring allows for the precise control of transcriptional activity necessary for stem cell pluripotency and differentiation. Moreover, the stability of chromatin ensures that stem cells can replicate their DNA faithfully and segregate chromosomes accurately during cell division, crucial for maintaining stem cell populations and preventing genomic instability that could lead to diseases like cancer. Accumulative evidence has demonstrated that phase separation plays a crucial role in mediating stem cell chromatin organization and stability. For instance, constitutive heterochromatin proteins, notably more disordered than other nucleome proteins, are implicated in heterochromatin formation through LLPS. Their expression, low initially, rises during preimplantation development, positioning the preimplantation embryo as a key model to explore LLPS in heterochromatin regulation. This highlights the potential for LLPS in managing chromatin states crucial for stem cell function and development [81]. Similarly, mouse heterochromatin undergoes phase separation early in embryogenesis, initially displaying liquid-like properties at the two-cell stage before maturing into a more structured, silent state by the four-cell stage. Disruption of these phase-separated compartments alters transcript levels, underscoring a pivotal role for phase separation in the organizational and functional dynamics of heterochromatin during development. This insight reveals how chromatin domains self-organize and transition during early mammalian embryogenesis [82]. Notably, MSR transcripts drive HP1α-mediated phase separation in ESCs, forming dynamic heterochromatin condensates essential for maintaining pluripotent nuclear architecture. Depletion of MSR transcripts results in more compact, static heterochromatin, leading to chromosome instability and mitotic defects. This demonstrates that MSR transcripts are crucial for modulating heterochromatin’s biophysical properties, highlighting their role in preserving genome stability through phase separation mechanisms [83]. **(**Table 1**)**

**Table 1:** The effect of phase separation on stem cell biology

Cell type	Regulation	Phase separation	Mechanism	Effects	Ref.
ESC	CINAP	Preventing NEDD4 from forming condensates	Decreasing YAP1 phosphorylation at Ser128 and YAP1 activation	Impacting endoderm differentiation of mESCs	[40]
ESC	Centrosome	Promoting LLPS of destruction complex	Enhancing processing of β-catenin	Promoting Wnt-driven ESC differentiation to mesoderm	[41]
mESC	RBD and IDR of LIN28A	Promoting phase-separated condensates of LIN28A and nucleolar proteins	Impacting dynamic nucleolar remodeling	Impacting naive-to-primed pluripotency state conversion	[42]
HSPC	/	Pabpc1b phase separation	Promoting Cpeb1b interaction and cytoplasmic polyadenylation of shha mRNA.	Impacting HSPC development	[43]
SSC	/	YTHDF1 LLPS	Activating the NF-κB-CCND1 axis	Promoting transdifferentiation of SSC to neural stem cell-like cell	[44]
MSC	SHP2E76K mutation	SHP2 LLPS	Leading to SHP2 dissociation from complexes I and III and complex I and III hyperactivation	Promoting malignant transformation from MSC to sarcoma stem-like cells	[45]
SSC	Upregulation of CBX2	CBX2 forming condensates	Regulating chromatin structure	Producing differentiating A1 spermatogonia	[51]
ESC	Polycomb	CBX7 Phase separation	Maintaining repressed chromatin	Impairing the ability of ESC to form embryoid bodies and neural progenitor cell	[52]
mESC	/	CBX7C and PHC2 form large, low-mobility chromatin-free aggregates	Facilitating PRC1 assembly	Regulating ESC differentiation	[53]
Pluripotent cell	IDR and N-terminal 70aa of SS18	SS18 forming microscopic condensates	Mediating BAF assembly	Regulating the transition from pluripotent to somatic states of stem cell	[54]
/	Sall4	Co-phase separation of Tet2/3 with Sall4 to form condensates	Mediating 5mC oxidation on bmp4 promoter, and enabling sufficient BMP signaling	Regulating pharyngeal cartilage development	[55]
Myogenic cells	/	Dact1 LLPS propensity to form Dact1 nuclear protein aggregates	Regulating Wnt signaling	Illustrating pathophysiological role in human muscular disease	[57]
Neural precursors	/	Prox1 LLPS	Recruiting and concentrating HP1 and inducing heterochromatin compaction	Guaranteeing terminal neuronal differentiation	[58]
hBMSC	/	YAP LLPS	Recruiting TAZ and TEAD4	Regulating osteogenic differentiation	[59]
ESC	IDR	Forming UTX phase-separated liquid condensates	Enhancing the H3K4 methylation activity of MLL4	Impacting ESC differentiation	[60]
Germline cell	MAGE-B2	Reducing G3BP protein levels and suppressing phase separation and SG initiation	Decreasing heat stress	Providing cytoprotection to maintain mammalian spermatogenesis	[61]
ESC	RYBP	CTCF phase separation	Promoting inter-A compartment interactions	Improving ESC self-renewal and inhibiting their differentiation toward neural progenitor cells	[65]
HSPC	ASXL1	Involving in paraspeckle formation	Upregulating NEAT1 expression and increasing NONO-NEAT1 interactions	Maintaining repopulating potential of HSPC	[66]
/	MIP-1 and MIP-2	Promoting P granule condensation	Recruiting and balancing essential RNA processing machinery	Regulating key developmental transitions in the germ line	[67]
/	LCD of ABCF1	ABCF1 phase separation	Coactivating OCT4/SOX2	Regulating stem cell self-renewal	[68]
Epiblast cell	Oct4	Highly expressed Oct4 alleles being closer to nuclear speckles	/	Regualting pluripotency transition	[69]
ESC	O-GlcNAcylation of Psme3	Decreasing P-body assembly	/	Maintenance of ESC pluripotent state	[70]
Haematopoietic stem cell	PPIA	Promoting LLPS	Increasing cellular stress resistance	Slowing down stem cell ageing	[75]
Mesenchymal progenitor cells	Arg 207 in SGF29	Promoting SGF29 condensates formation	Precise chromatin engagement Co-activator recruitment	Modulating transcriptional landscapes during aging	[76]
ISC	Ime4/ Mettl14	Promoting the formation of BuGZ condensation	Regulating MAPK pathway	Enhancing ISC proliferation Impacting gut repair and longevity	[77]
ESC	PML PML-NBs	Promoting TRIM33 puncta formation	Regulating Nodal signaling-directed transcription	Regulating nodal signaling in mESCs	[78]
ESC	PSPC1	Enhancing the formation of transcription condensates	Inhibiting the RNA-induced premature release of Pol II	Promoting polymerase binding and transcription	[79]
HSPC	/	Enhancing LLPS puncta of NUP98-HOXA9	Promoting the formation of transcription factor condensates	Inducing leukaemic transformation	[80]

## Therapeutic potential and clinical challenges of targeting phase separation

A growing body of research has highlighted the significant therapeutic potential of targeting phase separation in disease treatment, providing a robust theoretical framework for its clinical application. Moreover, the development of small-molecule inhibitors and translational strategies is currently being actively explored, further advancing this promising field. For instance, recent studies have revealed that disease-associated SHP2 mutants undergo LLPS, enhancing their phosphatase activity and promoting aberrant signaling pathways linked to various diseases, particularly developmental disorders and certain malignancies. The use of allosteric inhibitors to target SHP2-mediated LLPS shows increasing promise in treating various diseases, including cancers and developmental disorders. This therapeutic strategy is gaining growing recognition, and such targeted inhibitors are attracting expanding investment in research and development [84]. Similarly, in aged hematopoietic stem cells (HSCs), elevated levels of FUS—leading to aberrant phase transitions and reduced mobility—alter chromatin organization by merging topologically associating domains (TADs), thereby driving transcriptional changes linked to aging. These findings provide fresh insights into the molecular mechanisms underlying HSC aging and suggest that developing targeted inhibitors to modulate FUS phase behavior could offer novel therapeutic strategies for rejuvenating aged HSCs and treating age-related hematopoietic disorders [85]. In addition, the development of NoCasDrop—a tool enabling precise nucleolar targeting and controlled liquid condensation—marks a significant advancement in understanding and manipulating nucleolar phase separation dynamics. By restoring centromere clustering and perinucleolar heterochromatin (PNH) integrity, as well as regulating developmental gene expression, NoCasDrop effectively modulates stem cell pluripotency. This precise control over nucleolar condensation offers a potential therapeutic strategy for diseases associated with nucleolar dysfunction, such as certain cancers and developmental disorders. NoCasDrop exemplifies how targeted modulation of phase separation can influence chromatin organization and cell fate, providing new avenues for clinical interventions [86]. In regenerative medicine, LLPS has emerged as a promising target for enhancing tissue regeneration, particularly in aging tissues. Notably, 11,12-EET amplifies fibroblast growth factor signaling through phase separation, promoting the activation and proliferation of muscle stem cells. Preclinical studies have demonstrated that 11,12-EET, the representative bioactive lipid, acts as a tissue messenger to stimulate myogenesis. These insights highlight the potential of targeting LLPS mechanisms to develop innovative regenerative therapies, offering new avenues for clinical intervention in tissue repair and recovery [87]. In cancer biology, LLPS profoundly influences oncogenic processes and tumor cell survival. A notable example is fibrillarin (FBL), a nucleolar protein undergoing phase separation to promote acute myeloid leukemia (AML) cell survival by regulating pre-rRNA processing and oncogene translation. Targeting FBL phase separation domains with specific inhibitors, such as CGX-635, has demonstrate fficacy in eliminating AML cells, representing a novel therapeutic strategy [88]. Notably, pharmacological inhibition of core regulatory circuitry (CRC) transcription factors, such as HOXB8 and FOSL1, using the H3K27 demethylase inhibitor GSK-J4, has demonstrated significant therapeutic potential. GSK-J4 disrupts CRC condensates, leading to reduced tumor growth, suppression of metastasis, and restoration of chemosensitivity in patient-derived osteosarcoma models. This approach offers a promising strategy to provide new avenues for treating metastatic and chemoresistant osteosarcoma [89]. Collectively, these advancements underscore the therapeutic potential of targeting phase separation across a wide spectrum of diseases, paving the way for innovative clinical applications and transformative treatment strategies.

Recent advances in phase separation research have led to significant technological progress. Techniques such as live-cell super-resolution imaging, the combination of magnetic resonance and optical spectroscopies with molecular simulation have provided powerful tools to reveal the structure and dynamics of intracellular condensates [90–92]. In addition, machine learning-based predictors like PSPHunter and PSPire have deepened our understanding of the mechanisms underlying transcriptional regulation, cell fate transitions, and disease progression [93, 94]. Numerous preclinical studies have also demonstrated promising prospects for targeting phase separation as a therapeutic strategy [95].

Despite these promising therapeutic strategies and advancements, significant challenges remain in translating phase separation concepts into clinical applications. A major hurdle is the development of pharmacological agents capable of selectively modulating these dynamic structures. The current strategies involve directly disrupting phase separation via targeting IDRs or targeting components involved in phase separation [96]. However, unlike stable protein complexes, phase-separated condensates lack rigid architecture, rendering conventional drug design approaches inadequate. Most current drug targets are enzymes, ion channels, G-protein-coupled receptors, kinases, nuclear receptors, and transporters, while over half of the genome, including intrinsically disordered proteins (IDPs), does not fall into these categories [97, 98]. The physicochemical principles of LLPS, which underpin condensate formation, offer a novel conceptual framework for small molecule interactions with IDPs—one that does not necessarily involve well-defined binding sites but rather physicochemical mechanisms. However, identifying such molecules requires carefully designed compound libraries and a reevaluation of what constitutes a drug-like small molecule. Furthermore, it remains uncertain whether targeting physicochemical mechanisms will yield sufficient therapeutic efficacy and specificity [99]. Addressing this challenge requires innovative strategies to manipulate the physicochemical properties of condensates, including controlling intermolecular forces and modulating post-translational modifications that govern their formation and dissolution.

In addition, the specific mechanisms of LLPS in various diseases and the complexity of the associated signaling pathways remain poorly understood. The safety and efficacy of drugs targeting phase separation in complex physiological environments also require thorough evaluation, as many inhibitors have yet to undergo clinical trials. Advanced imaging technologies and organoid culture techniques offer promising solutions to these challenges, facilitating the clinical translation of phase separation-targeted therapies. These models are capable of capturing the kinetics of condensate dynamics under both physiological and pathological conditions. Nonetheless, further efforts are needed to develop models that more accurately recapitulate the complex in vivo environment and to conduct clinical trials to evaluate the safety and efficacy of LLPS inhibitors [100]. Furthermore, the development of biomarkers to monitor condensate behavior in real time will be crucial for assessing therapeutic responses. Achieving clinical success will require interdisciplinary collaborations that seamlessly integrate molecular biology, biophysics, medicinal chemistry, and clinical research, transforming fundamental discoveries into practical and clinically relevant therapies [101].

## Conclusions and future perspectives

Phase separation plays a pivotal role in stem cell biology, offering profound insights into cellular organization and function. This phenomenon, crucial for the spatial and temporal regulation of biological activities, holds significant promise for advancing stem cell-based therapies, potentially revolutionizing treatments for a wide range of diseases. However, translating these insights into practical applications presents considerable challenges.

Firstly, manipulating biological phase separation offers significant potential for enhancing stem cell therapies in disease treatment [89]. However, current research on regulating the physiological functions of stem cells through phase separation remains sparse. There is a notable deficiency in stable and reliable molecular targets and therapeutics associated with this approach. Addressing this gap necessitates a concerted effort to identify and characterize molecular interactions and pathways that govern phase separation in stem cells. This deeper understanding could lead to the development of novel therapeutic strategies that are both effective and precise, potentially revolutionizing the field of regenerative medicine by enabling targeted manipulation of stem cell behaviors at the molecular level.

Secondly, despite considerable progress in the field of stem cell biology, particularly in the understanding of phase separation, the majority of current research conducted in vitro might not fully reflect the intricate dynamics of the in vivo environment [102–104]. This gap highlights the need for more innovative methodologies that enable real-time, in situ observation and manipulation of phase separation within living organisms [105]. Moreover, existing microscopy techniques, which are crucial for studying these phenomena, currently face significant limitations in terms of resolution, dynamic range, and labeling methods. There is a critical demand for the development of advanced imaging technologies that can offer higher resolution and better spatiotemporal capabilities to capture detailed processes. Additionally, phase separation is a highly dynamic and reversible process, influenced by a range of physiological and environmental factors [21, 106, 107]. It is characterized by rapid transitions that can occur within seconds to minutes, presenting substantial technical challenges in tracking and analyzing the behavior of individual molecules. These complexities make it particularly difficult to effectively capture and study these transient phenomena, which are crucial for a deeper understanding of cellular function and dysfunction.

Thirdly, phase separation encompasses a wide range of biomolecules, including proteins, RNA, and metabolites, each contributing significantly to various biological functions [108]. The complex interactions among these molecules represent a critical focal point in the field, introducing substantial challenges for both fundamental research and therapeutic application. The intricate nature of these interactions necessitates a comprehensive understanding to harness the therapeutic potential of phase separation effectively. Precisely modulating these processes to treat diseases requires sophisticated techniques that can capture and manipulate the dynamic, often transient states of phase separation. Achieving this level of control demands a robust integration of biophysical, biochemical, and technological insights, which are essential for developing effective therapeutic strategies.

Lastly, the individual genetic background significantly influences the behavior of stem cells derived from different sources, particularly in their response to phase separation processes. This variability underscores the critical importance of personalized medicine in the context of stem cell-based therapy [109, 110]. As phase separation plays a pivotal role in cellular functions and disease progression, understanding how individual genetic differences affect these mechanisms is essential [111]. Tailoring treatments to the specific genetic and cellular context of each patient can enhance the efficacy of stem cell-based interventions.

## Conclusions

Phase separation plays a crucial role in stem cell biology, influencing cellular organization, disease progression, and therapeutic applications. Its dynamic and complex nature is essential for regulating key biological processes. To harness the therapeutic potential of phase separation, advancements in imaging and analytical techniques are necessary, along with a deeper molecular understanding of phase separation mechanisms Future research should focus on elucidating the interplay between phase separation and stem cell biology, aiming to develop novel therapeutic strategies. Such efforts promise to lead to more effective treatments for a variety of diseases, marking significant advancements in medical science and therapeutic interventions.

## Data Availability

Not applicable.

## Abbreviations

TCR: T cell receptors
ESCs: Embryonic stem cells
MSCs: Mesenchymal stem cells
LLPS: Liquid-liquid phase separation
SGs: Stress granules
PTMs: Post-translational modifications
CSC: Cancer stem cell
CBX: Chromobox
PRC1: Polycomb repressive complex 1
PST: Pluripotent to somatic transition
IDR: Intrinsically disordered region
HSPCs: Hematopoietic stem and progenitor cells
PPIA: Peptidyl-prolyl isomerase A
ISCs: Intestinal stem cells

## References

[1] Banani SF, Lee HO, Hyman AA, Rosen MK. Biomolecular condensates: organizers of cellular biochemistry. Nat Rev Mol Cell Biol. 2017;18(5):285–98. 28225081 10.1038/nrm.2017.7 PMC7434221

[2] Hyman AA, Weber CA, Julicher F. Liquid-liquid phase separation in biology. Annu Rev Cell Dev Biol. 2014;30:39–58. 25288112 10.1146/annurev-cellbio-100913-013325

[3] Wheeler JR, Matheny T, Jain S, Abrisch R, Parker R. Distinct stages in stress granule assembly and disassembly. Elife. 2016;5. 10.7554/eLife.18413 PMC5014549 27602576

[4] Tong X, Tang R, Xu J, Wang W, Zhao Y, Yu X, et al. Liquid-liquid phase separation in tumor biology. Signal Transduct Target Ther. 2022;7(1):221. 35803926 10.1038/s41392-022-01076-x PMC9270353

[5] Zhao YG, Zhang H. Phase separation in membrane biology: the interplay between membrane-Bound organelles and membraneless condensates. Dev Cell. 2020;55(1):30–44. 32726575 10.1016/j.devcel.2020.06.033

[6] Gao Y, Li X, Li P, Lin Y. A brief guideline for studies of phase-separated biomolecular condensates. Nat Chem Biol. 2022;18(12):1307–18. 36400991 10.1038/s41589-022-01204-2

[7] Boyd-Shiwarski CR, Shiwarski DJ, Griffiths SE, Beacham RT, Norrell L, Morrison DE, et al. WNK kinases sense molecular crowding and rescue cell volume via phase separation. Cell. 2022;185(24):4488–506. e20. 36318922 10.1016/j.cell.2022.09.042 PMC9699283

[8] Chen H, Xu X, Hu W, Wu S, Xiao J, Wu P, et al. Self-programmed dynamics of T cell receptor condensation. Proc Natl Acad Sci U S A. 2023;120(28):e2217301120. 37399423 10.1073/pnas.2217301120 PMC10334747

[9] Sun Z, Yu H, Zhao J, Tan T, Pan H, Zhu Y, et al. LIN28 coordinately promotes nucleolar/ribosomal functions and represses the 2 C-like transcriptional program in pluripotent stem cells. Protein Cell. 2022;13(7):490–512. 34331666 10.1007/s13238-021-00864-5 PMC9226220

[10] Lin Y, Zheng J, Mai Z, Lin P, Lu Y, Cui L, et al. Unveiling the veil of RNA binding protein phase separation in cancer biology and therapy. Cancer Lett. 2024;601:217160. 39111384 10.1016/j.canlet.2024.217160

[11] Maccaroni K, La Torre M, Burla R, Saggio I. Phase separation in the nucleus and at the nuclear periphery during Post-Mitotic nuclear envelope reformation. Cells. 2022;11(11). 10.3390/cells11111749 PMC9179440 35681444

[12] Yang P, Mathieu C, Kolaitis RM, Zhang P, Messing J, Yurtsever U, et al. G3BP1 is a tunable switch that triggers phase separation to assemble stress granules. Cell. 2020;181(2):325–45. e28. 32302571 10.1016/j.cell.2020.03.046 PMC7448383

[13] Barrow ER, Valionyte E, Baxter CR, Yang Y, Herath S, O’Connell WA, et al. Discovery of SQSTM1/p62-dependent P-bodies that regulate the NLRP3 inflammasome. Cell Rep. 2024;43(3):113935. 38460129 10.1016/j.celrep.2024.113935

[14] Xu B, Huang S, Liu Y, Wan C, Gu Y, Wang D, et al. Manganese promotes alpha-synuclein amyloid aggregation through the induction of protein phase transition. J Biol Chem. 2022;298(1):101469. 34871547 10.1016/j.jbc.2021.101469 PMC8717548

[15] Feng Z, Jia B, Zhang M. Liquid-Liquid phase separation in biology: specific stoichiometric molecular interactions vs promiscuous interactions mediated by disordered sequences. Biochemistry. 2021;60(31):2397–406. 34291921 10.1021/acs.biochem.1c00376

[16] Martin EW, Holehouse AS. Intrinsically disordered protein regions and phase separation: sequence determinants of assembly or lack thereof. Emerg Top Life Sci. 2020;4(3):307–29. 33078839 10.1042/ETLS20190164

[17] Soding J, Zwicker D, Sohrabi-Jahromi S, Boehning M, Kirschbaum J. Mechanisms for active regulation of biomolecular condensates. Trends Cell Biol. 2020;30(1):4–14. 31753533 10.1016/j.tcb.2019.10.006

[18] Sabari BR, Dall’Agnese A, Boija A, Klein IA, Coffey EL, Shrinivas K et al. Coactivator condensation at super-enhancers links phase separation and gene control. Science. 2018;361(6400). 10.1126/science.aar3958 PMC6092193 29930091

[19] Shin Y, Brangwynne CP. Liquid phase condensation in cell physiology and disease. Science. 2017;357(6357). 10.1126/science.aaf4382 28935776

[20] Murthy AC, Dignon GL, Kan Y, Zerze GH, Parekh SH, Mittal J, et al. Molecular interactions underlying liquid-liquid phase separation of the FUS low-complexity domain. Nat Struct Mol Biol. 2019;26(7):637–48. 31270472 10.1038/s41594-019-0250-x PMC6613800

[21] Zhang W, Li Z, Wang X, Sun T. Phase separation is regulated by post-translational modifications and participates in the developments of human diseases. Heliyon. 2024;10(13):e34035. 39071719 10.1016/j.heliyon.2024.e34035 PMC11279762

[22] Li J, Zhang M, Ma W, Yang B, Lu H, Zhou F, et al. Post-translational modifications in liquid-liquid phase separation: a comprehensive review. Mol Biomed. 2022;3(1):13. 35543798 10.1186/s43556-022-00075-2 PMC9092326

[23] Yamazaki H, Takagi M, Kosako H, Hirano T, Yoshimura SH. Cell cycle-specific phase separation regulated by protein charge blockiness. Nat Cell Biol. 2022;24(5):625–32. 35513709 10.1038/s41556-022-00903-1 PMC9106583

[24] Wei M, Huang X, Liao L, Tian Y, Zheng X. SENP1 decreases RNF168 phase separation to promote DNA damage repair and drug resistance in Colon cancer. Cancer Res. 2023;83(17):2908–23. 37350666 10.1158/0008-5472.CAN-22-4017

[25] Sanchez-Burgos I, Espinosa JR, Joseph JA, Collepardo-Guevara R. Valency and binding affinity variations can regulate the multilayered organization of protein condensates with many components. Biomolecules. 2021;11(2). 10.3390/biom11020278 PMC7918469 33672806

[26] Jankowsky E, Harris ME. Specificity and nonspecificity in RNA-protein interactions. Nat Rev Mol Cell Biol. 2015;16(9):533–44. 26285679 10.1038/nrm4032 PMC4744649

[27] Xin D, Gai X, Ma Y, Li Z, Li Q, Yu X. Pre-rRNA facilitates TopBP1-Mediated DNA Double-Strand break response. Adv Sci (Weinh). 2023;10(28):e2206931. 37582658 10.1002/advs.202206931 PMC10558638

[28] Li RH, Tian T, Ge QW, He XY, Shi CY, Li JH, et al. A phosphatidic acid-binding LncRNA SNHG9 facilitates LATS1 liquid-liquid phase separation to promote oncogenic YAP signaling. Cell Res. 2021;31(10):1088–105. 34267352 10.1038/s41422-021-00530-9 PMC8486796

[29] Bergeron-Sandoval LP, Safaee N, Michnick SW. Mechanisms and consequences of macromolecular phase separation. Cell. 2016;165(5):1067–79. 27203111 10.1016/j.cell.2016.05.026

[30] Liu Q, Li J, Zhang W, Xiao C, Zhang S, Nian C, et al. Glycogen accumulation and phase separation drives liver tumor initiation. Cell. 2021;184(22):5559–e7619. 34678143 10.1016/j.cell.2021.10.001

[31] Liu MJ, Yeh FJ, Yvon R, Simpson K, Jordan S, Chambers J, et al. Extracellular pectin-RALF phase separation mediates FERONIA global signaling function. Cell. 2024;187(2):312–30. e22. 38157854 10.1016/j.cell.2023.11.038

[32] Tessier S, Ferhi O, Geoffroy MC, Gonzalez-Prieto R, Canat A, Quentin S, et al. Exploration of nuclear body-enhanced sumoylation reveals that PML represses 2-cell features of embryonic stem cells. Nat Commun. 2022;13(1):5726. 36175410 10.1038/s41467-022-33147-6 PMC9522831

[33] Shi B, Heng J, Zhou JY, Yang Y, Zhang WY, Koziol MJ, et al. Phase separation of Ddx3xb helicase regulates maternal-to-zygotic transition in zebrafish. Cell Res. 2022;32(8):715–28. 35661831 10.1038/s41422-022-00655-5 PMC9343644

[34] Mensah MA, Niskanen H, Magalhaes AP, Basu S, Kircher M, Sczakiel HL, et al. Aberrant phase separation and nucleolar dysfunction in rare genetic diseases. Nature. 2023;614(7948):564–71. 36755093 10.1038/s41586-022-05682-1 PMC9931588

[35] Boyko S, Surewicz WK. Tau liquid-liquid phase separation in neurodegenerative diseases. Trends Cell Biol. 2022;32(7):611–23. 35181198 10.1016/j.tcb.2022.01.011 PMC9189016

[36] Taniue K, Akimitsu N. Aberrant phase separation and cancer. FEBS J. 2022;289(1):17–39. 33583140 10.1111/febs.15765

[37] Mo Y, Feng Y, Huang W, Tan N, Li X, Jie M, et al. Liquid-Liquid phase separation in cardiovascular diseases. Cells. 2022;11:19. 10.3390/cells11193040 PMC9562871 36231002

[38] Ye J, Huang X, Yuan M, Wang J, Jia R, Wang T et al. HSD17B13 liquid-liquid phase separation promotes leukocyte adhesion in chronic liver inflammation. J Mol Cell Biol. 2024. 10.1093/jmcb/mjae018 PMC11631211 38692847

[39] Wang J, Zhou Y, Zhang M, Wu Y, Wu Q, Su W, et al. YTHDF1-CLOCK axis contributes to pathogenesis of allergic airway inflammation through LLPS. Cell Rep. 2024;43(3):113947. 38492220 10.1016/j.celrep.2024.113947

[40] Zhuge R, Wang C, Wang J, Yu S, Liao L, Zheng X. hCINAP regulates the differentiation of embryonic stem cells by regulating NEDD4 liquid-liquid phase-separation-mediated YAP1 activation. Cell Rep. 2023;42(1):111935. 36640330 10.1016/j.celrep.2022.111935

[41] Lach RS, Qiu C, Kajbaf EZ, Baxter N, Han D, Wang A, et al. Nucleation of the destruction complex on the centrosome accelerates degradation of beta-catenin and regulates Wnt signal transmission. Proc Natl Acad Sci U S A. 2022;119(36):e2204688119. 36037369 10.1073/pnas.2204688119 PMC9457612

[42] Tan T, Gao B, Yu H, Pan H, Sun Z, Lei A, et al. Dynamic nucleolar phase separation influenced by non-canonical function of LIN28A instructs pluripotent stem cell fate decisions. Nat Commun. 2024;15(1):1256. 38341436 10.1038/s41467-024-45451-4 PMC10858886

[43] Heng J, Shi B, Zhou JY, Zhang Y, Ma D, Yang YG, et al. Cpeb1b-mediated cytoplasmic polyadenylation of Shha mRNA modulates zebrafish definitive hematopoiesis. Proc Natl Acad Sci U S A. 2023;120(7):e2212212120. 36745802 10.1073/pnas.2212212120 PMC9964029

[44] Fang Q, Tian GG, Wang Q, Liu M, He L, Li S, et al. YTHDF1 phase separation triggers the fate transition of spermatogonial stem cells by activating the IkappaB-NF-kappaB-CCND1 axis. Cell Rep. 2023;42(4):112403. 37060562 10.1016/j.celrep.2023.112403

[45] Kan C, Tan Z, Liu L, Liu B, Zhan L, Zhu J et al. Phase separation of SHP2E76K promotes malignant transformation of mesenchymal stem cells by activating mitochondrial complexes. JCI Insight. 2024;9(8). 10.1172/jci.insight.170340 PMC11141883 38451719

[46] Burkart RC, Strotmann VI, Kirschner GK, Akinci A, Czempik L, Dolata A, et al. PLETHORA-WOX5 interaction and subnuclear localization control Arabidopsis root stem cell maintenance. EMBO Rep. 2022;23(6):e54105. 35373503 10.15252/embr.202154105 PMC9171415

[47] Chera S, Rentzsch F. Stem cells: the cell that does it all. Curr Biol. 2023;33(11):R434–6. 37279662 10.1016/j.cub.2023.04.039

[48] Bonello TT, Cai D, Fletcher GC, Wiengartner K, Pengilly V, Lange KS, et al. Phase separation of Hippo signalling complexes. EMBO J. 2023;42(6):e112863. 36807601 10.15252/embj.2022112863 PMC10015380

[49] Li Q, Gao P. Phase separation in cGAS-STING signaling. Front Med. 2023;17(5):855–66. 37906339 10.1007/s11684-023-1026-6

[50] Esposito M, Fang C, Cook KC, Park N, Wei Y, Spadazzi C, et al. TGF-beta-induced DACT1 biomolecular condensates repress Wnt signalling to promote bone metastasis. Nat Cell Biol. 2021;23(3):257–67. 33723425 10.1038/s41556-021-00641-w PMC7970447

[51] Kim JJ, Steinson ER, Lau MS, de Rooij DG, Page DC, Kingston RE. Cell type-specific role of CBX2 and its disordered region in spermatogenesis. Genes Dev. 2023;37(13–14):640–60. 37553262 10.1101/gad.350393.122 PMC10499018

[52] Jaensch ES, Zhu J, Cochrane JC, Marr SK, Oei TA, Damle M, et al. A polycomb domain found in committed cells impairs differentiation when introduced into PRC1 in pluripotent cells. Mol Cell. 2021;81(22):4677–e918. 34637753 10.1016/j.molcel.2021.09.018 PMC8966356

[53] Guan S, Tang J, Ma X, Miao R, Cheng B. CBX7C⋅PHC2 interaction facilitates PRC1 assembly and modulates its phase separation properties. iScience. 2024;27(4):109548. 38600974 10.1016/j.isci.2024.109548 PMC11004992

[54] Kuang J, Zhai Z, Li P, Shi R, Guo W, Yao Y, et al. SS18 regulates pluripotent-somatic transition through phase separation. Nat Commun. 2021;12(1):4090. 34215745 10.1038/s41467-021-24373-5 PMC8253816

[55] Wang W, Yang N, Wang L, Zhu Y, Chu X, Xu W, et al. The TET-Sall4-BMP regulatory axis controls craniofacial cartilage development. Cell Rep. 2024;43(3):113873. 38427557 10.1016/j.celrep.2024.113873

[56] Tripathi S, Miyake T, Kelebeev J, McDermott JC. TAZ exhibits phase separation properties and interacts with Smad7 and beta-catenin to repress skeletal myogenesis. J Cell Sci. 2022;135(1). 10.1242/jcs.259097 34859820

[57] Contriciani RE, da Veiga FC, do Amaral MJ, Castelucci BG, de Sousa LM, de Jesus MB, et al. Dact1 is expressed during chicken and mouse skeletal myogenesis and modulated in human muscle diseases. Comp Biochem Physiol B Biochem Mol Biol. 2021;256:110645. 34252542 10.1016/j.cbpb.2021.110645

[58] Liu X, Shen J, Xie L, Wei Z, Wong C, Li Y, et al. Mitotic implantation of the transcription factor prospero via phase separation drives terminal neuronal differentiation. Dev Cell. 2020;52(3):277–93. e8. 31866201 10.1016/j.devcel.2019.11.019

[59] Tan K, Yang Q, Han Y, Zhuang Z, Zhao Y, Guo K, et al. Elastic modulus of hydrogel regulates osteogenic differentiation via liquid-liquid phase separation of YAP. J Biomed Mater Res A. 2023;111(11):1781–97. 37494632 10.1002/jbm.a.37590

[60] Shi B, Li W, Song Y, Wang Z, Ju R, Ulman A, et al. UTX condensation underlies its tumour-suppressive activity. Nature. 2021;597(7878):726–31. 34526716 10.1038/s41586-021-03903-7 PMC9008583

[61] Lee AK, Klein J, Fon Tacer K, Lord T, Oatley MJ, Oatley JM, et al. Translational repression of G3BP in Cancer and germ cells suppresses stress granules and enhances stress tolerance. Mol Cell. 2020;79(4):645–59. e9. 32692974 10.1016/j.molcel.2020.06.037

[62] Wang J, Yu H, Ma Q, Zeng P, Wu D, Hou Y, et al. Phase separation of OCT4 controls TAD reorganization to promote cell fate transitions. Cell Stem Cell. 2021;28(10):1868–e8311. 34038708 10.1016/j.stem.2021.04.023

[63] Gardner RL. Stem cells: potency, plasticity and public perception. J Anat. 2002;200(Pt 3):277–82. 12033732 10.1046/j.1469-7580.2002.00029.x PMC1570679

[64] Zakrzewski W, Dobrzynski M, Szymonowicz M, Rybak Z. Stem cells: past, present, and future. Stem Cell Res Ther. 2019;10(1):68. 30808416 10.1186/s13287-019-1165-5 PMC6390367

[65] Wei C, Jia L, Huang X, Tan J, Wang M, Niu J, et al. CTCF organizes inter-A compartment interactions through RYBP-dependent phase separation. Cell Res. 2022;32(8):744–60. 35768498 10.1038/s41422-022-00676-0 PMC9343660

[66] Yamamoto K, Goyama S, Asada S, Fujino T, Yonezawa T, Sato N, et al. A histone modifier, ASXL1, interacts with NONO and is involved in paraspeckle formation in hematopoietic cells. Cell Rep. 2021;36(8):109576. 34433054 10.1016/j.celrep.2021.109576

[67] Cipriani PG, Bay O, Zinno J, Gutwein M, Gan HH, Mayya VK et al. Novel LOTUS-domain proteins are organizational hubs that recruit C. elegans Vasa to germ granules. Elife. 2021;10. 10.7554/eLife.60833 PMC8331183 34223818

[68] Choi EB, Vodnala M, Zerbato M, Wang J, Ho JJ, Inouye C, et al. ATP-binding cassette protein ABCF1 couples transcription and genome surveillance in embryonic stem cells through low-complexity domain. Sci Adv. 2021;7(44):eabk2775. 34714667 10.1126/sciadv.abk2775 PMC8555894

[69] Garate X, Gomez-Garcia PA, Merino MF, Angles MC, Zhu C, Castells-Garcia A, et al. The relationship between nanoscale genome organization and gene expression in mouse embryonic stem cells during pluripotency transition. Nucleic Acids Res. 2024;52(14):8146–64. 38850157 10.1093/nar/gkae476 PMC11317139

[70] Pecori F, Kondo N, Ogura C, Miura T, Kume M, Minamijima Y, et al. Site-specific O-GlcNAcylation of Psme3 maintains mouse stem cell pluripotency by impairing P-body homeostasis. Cell Rep. 2021;36(2):109361. 34260942 10.1016/j.celrep.2021.109361

[71] Brunet A, Goodell MA, Rando TA. Ageing and rejuvenation of tissue stem cells and their niches. Nat Rev Mol Cell Biol. 2023;24(1):45–62. 35859206 10.1038/s41580-022-00510-w PMC9879573

[72] Liu B, Qu J, Zhang W, Izpisua Belmonte JC, Liu GH. A stem cell aging framework, from mechanisms to interventions. Cell Rep. 2022;41(3):111451. 36261013 10.1016/j.celrep.2022.111451

[73] Jamieson CHM, Weissman IL. Stem-Cell aging and pathways to precancer evolution. N Engl J Med. 2023;389(14):1310–9. 37792614 10.1056/NEJMra2304431 PMC11908798

[74] Cesselli D, Aleksova A, Mazzega E, Caragnano A, Beltrami AP. Cardiac stem cell aging and heart failure. Pharmacol Res. 2018;127:26–32. 28111264 10.1016/j.phrs.2017.01.013

[75] Maneix L, Iakova P, Lee CG, Moree SE, Lu X, Datar GK, et al. Cyclophilin A supports translation of intrinsically disordered proteins and affects Haematopoietic stem cell ageing. Nat Cell Biol. 2024;26(4):593–603. 38553595 10.1038/s41556-024-01387-x PMC11021199

[76] Yan K, Ji Q, Zhao D, Li M, Sun X, Wang Z, et al. SGF29 nuclear condensates reinforce cellular aging. Cell Discov. 2023;9(1):110. 37935676 10.1038/s41421-023-00602-7 PMC10630320

[77] Zhang Q, Deng K, Liu M, Yang S, Xu W, Feng T, et al. Phase separation of BuGZ regulates gut regeneration and aging through interaction with m(6)A regulators. Nat Commun. 2023;14(1):6700. 37872148 10.1038/s41467-023-42474-1 PMC10593810

[78] Sun H, Chen Y, Yan K, Shao Y, Zhang QC, Lin Y, et al. Recruitment of TRIM33 to cell-context specific PML nuclear bodies regulates nodal signaling in mESCs. EMBO J. 2023;42(3):e112058. 36524443 10.15252/embj.2022112058 PMC9890237

[79] Shao W, Bi X, Pan Y, Gao B, Wu J, Yin Y, et al. Phase separation of RNA-binding protein promotes polymerase binding and transcription. Nat Chem Biol. 2022;18(1):70–80. 34916619 10.1038/s41589-021-00904-5

[80] Ahn JH, Davis ES, Daugird TA, Zhao S, Quiroga IY, Uryu H, et al. Phase separation drives aberrant chromatin looping and cancer development. Nature. 2021;595(7868):591–5. 34163069 10.1038/s41586-021-03662-5 PMC8647409

[81] Guthmann M, Burton A, Torres-Padilla ME. Expression and phase separation potential of heterochromatin proteins during early mouse development. EMBO Rep. 2019;20(12):e47952. 31701657 10.15252/embr.201947952 PMC6893284

[82] Guthmann M, Qian C, Gialdini I, Nakatani T, Ettinger A, Schauer T, et al. A change in biophysical properties accompanies heterochromatin formation in mouse embryos. Genes Dev. 2023;37(7–8):336–50. 37072228 10.1101/gad.350353.122 PMC10153458

[83] Novo CL, Wong EV, Hockings C, Poudel C, Sheekey E, Wiese M, et al. Satellite repeat transcripts modulate heterochromatin condensates and safeguard chromosome stability in mouse embryonic stem cells. Nat Commun. 2022;13(1):3525. 35725842 10.1038/s41467-022-31198-3 PMC9209518

[84] Zhu G, Xie J, Kong W, Xie J, Li Y, Du L, et al. Phase separation of Disease-Associated SHP2 mutants underlies MAPK hyperactivation. Cell. 2020;183(2):490–502. e18. 33002410 10.1016/j.cell.2020.09.002 PMC7572904

[85] Tang B, Wang X, He H, Chen R, Qiao G, Yang Y, et al. Aging-disturbed FUS phase transition impairs hematopoietic stem cells by altering chromatin structure. Blood. 2024;143(2):124–38. 37748139 10.1182/blood.2023020539

[86] Shi X, Li Y, Zhou H, Hou X, Yang J, Malik V, et al. DDX18 coordinates nucleolus phase separation and nuclear organization to control the pluripotency of human embryonic stem cells. Nat Commun. 2024;15(1):10803. 39738032 10.1038/s41467-024-55054-8 PMC11685540

[87] Chi Z, Chen S, Yang D, Cui W, Lu Y, Wang Z, et al. Gasdermin D-mediated metabolic crosstalk promotes tissue repair. Nature. 2024;634(8036):1168–77. 39260418 10.1038/s41586-024-08022-7

[88] Yang L, Zhang Z, Jiang P, Kong D, Yu Z, Shi D, et al. Phase separation-competent FBL promotes early pre-rRNA processing and translation in acute myeloid leukaemia. Nat Cell Biol. 2024;26(6):946–61. 38745030 10.1038/s41556-024-01420-z

[89] Lu B, Zou C, Yang M, He Y, He J, Zhang C, et al. Pharmacological Inhibition of core regulatory circuitry Liquid-liquid phase separation suppresses metastasis and chemoresistance in osteosarcoma. Adv Sci (Weinh). 2021;8(20):e2101895. 34432948 10.1002/advs.202101895 PMC8529446

[90] Du M, Stitzinger SH, Spille JH, Cho WK, Lee C, Hijaz M, et al. Direct observation of a condensate effect on super-enhancer controlled gene bursting. Cell. 2024;187(2):331–44. e17. 38194964 10.1016/j.cell.2023.12.005

[91] Fawzi NL, Parekh SH, Mittal J. Biophysical studies of phase separation integrating experimental and computational methods. Curr Opin Struct Biol. 2021;70:78–86. 34144468 10.1016/j.sbi.2021.04.004 PMC8530909

[92] Liu Z, Qin Z, Liu Y, Xia X, He L, Chen N, et al. Liquid–liquid phase separation: roles and implications in future cancer treatment. Int J Biol Sci. 2023;19(13):4139–56. 37705755 10.7150/ijbs.81521 PMC10496506

[93] Hou S, Hu J, Yu Z, Li D, Liu C, Zhang Y. Machine learning predictor PSPire screens for phase-separating proteins lacking intrinsically disordered regions. Nat Commun. 2024;15(1):2147. 38459060 10.1038/s41467-024-46445-y PMC10923898

[94] Sun J, Qu J, Zhao C, Zhang X, Liu X, Wang J, et al. Precise prediction of phase-separation key residues by machine learning. Nat Commun. 2024;15(1):2662. 38531854 10.1038/s41467-024-46901-9 PMC10965946

[95] Wang B, Zhang L, Dai T, Qin Z, Lu H, Zhang L, et al. Liquid-liquid phase separation in human health and diseases. Signal Transduct Target Ther. 2021;6(1):290. 34334791 10.1038/s41392-021-00678-1 PMC8326283

[96] Zheng LW, Liu CC, Yu KD. Phase separations in oncogenesis, tumor progressions and metastasis: a glance from hallmarks of cancer. J Hematol Oncol. 2023;16(1):123. 38110976 10.1186/s13045-023-01522-5 PMC10726551

[97] Santos R, Ursu O, Gaulton A, Bento AP, Donadi RS, Bologa CG, et al. A comprehensive map of molecular drug targets. Nat Rev Drug Discov. 2017;16(1):19–34. 27910877 10.1038/nrd.2016.230 PMC6314433

[98] Wang F, Zhang Y. Physiology and Pharmacological targeting of phase separation. J Biomed Sci. 2024;31(1):11. 38245749 10.1186/s12929-024-00993-z PMC10800077

[99] Wheeler RJ. Therapeutics-how to treat phase separation-associated diseases. Emerg Top Life Sci. 2020;4(3):307–18. 32364240 10.1042/ETLS20190176 PMC7733670

[100] Zou Y, Zheng H, Ning Y, Yang Y, Wen Q, Fan S. New insights into the important roles of phase seperation in the targeted therapy of lung cancer. Cell Biosci. 2023;13(1):150. 37580790 10.1186/s13578-023-01101-8 PMC10426191

[101] Boeynaems S, Chong S, Gsponer J, Holt L, Milovanovic D, Mitrea DM, et al. Phase separation in biology and disease; current perspectives and open questions. J Mol Biol. 2023;435(5):167971. 36690068 10.1016/j.jmb.2023.167971 PMC9970028

[102] Munda M, Velnar T. Stem cell therapy for degenerative disc disease: bridging the gap between preclinical promise and clinical potential. Biomol Biomed. 2024;24(2):210–8. 37669102 10.17305/bb.2023.9518 PMC10950333

[103] Sakthiswary R, Raymond AA. Stem cell therapy in neurodegenerative diseases: from principles to practice. Neural Regen Res. 2012;7(23):1822–31. 25624807 10.3969/j.issn.1673-5374.2012.23.009 PMC4302533

[104] Cha Z, Qiao Y, Lu Q, Wang Q, Lu X, Zhou H, et al. Research progress and challenges of stem cell therapy for ischemic stroke. Front Cell Dev Biol. 2024;12:1410732. 39040041 10.3389/fcell.2024.1410732 PMC11260720

[105] Mittag T, Pappu RV. A conceptual framework for Understanding phase separation and addressing open questions and challenges. Mol Cell. 2022;82(12):2201–14. 35675815 10.1016/j.molcel.2022.05.018 PMC9233049

[106] Xiao Q, McAtee CK, Su X. Phase separation in immune signalling. Nat Rev Immunol. 2022;22(3):188–99. 34230650 10.1038/s41577-021-00572-5 PMC9674404

[107] Nott TJ, Petsalaki E, Farber P, Jervis D, Fussner E, Plochowietz A, et al. Phase transition of a disordered Nuage protein generates environmentally responsive membraneless organelles. Mol Cell. 2015;57(5):936–47. 25747659 10.1016/j.molcel.2015.01.013 PMC4352761

[108] Luo H, Kharas MG. Dotting out AML by targeting fibrillarin. Cancer Res. 2024;84(17):2759–60. 38924716 10.1158/0008-5472.CAN-24-2125 PMC12182715

[109] Desgres M, Menasche P. Clinical translation of pluripotent stem cell therapies: challenges and considerations. Cell Stem Cell. 2019;25(5):594–606. 31703770 10.1016/j.stem.2019.10.001

[110] Yamanaka S. Pluripotent stem cell-Based cell Therapy-Promise and challenges. Cell Stem Cell. 2020;27(4):523–31. 33007237 10.1016/j.stem.2020.09.014

[111] Alberti S, Dormann D. Liquid-Liquid phase separation in disease. Annu Rev Genet. 2019;53:171–94. 31430179 10.1146/annurev-genet-112618-043527

